# Vaccination with Mincle agonist UM-1098 and mycobacterial antigens induces protective Th1 and Th17 responses

**DOI:** 10.1038/s41541-024-00897-x

**Published:** 2024-06-06

**Authors:** Viktoria Rungelrath, Mushtaq Ahmed, Linda Hicks, Shannon M. Miller, Kendal T. Ryter, Kyle Montgomery, George Ettenger, Alexander Riffey, Walid M. Abdelwahab, Shabaana Abdul Khader, Jay T. Evans

**Affiliations:** 1 Center for Translational Medicine, University of Montana, 32 Campus Drive, Missoula, MT 59812 USA; 2 Department of Biomedical & Pharmaceutical Sciences, University of Montana, Missoula, MT 59812 USA; 3 Department of Microbiology, University of Chicago, 920 E. 58th St., Chicago, IL 60637 USA

**Keywords:** Adjuvants, Vaccines

## Abstract

Tuberculosis (TB), caused by *Mycobacterium tuberculosis (Mtb)*, is one of the top infectious killers in the world. The only licensed vaccine against TB, Bacille Calmette-Guérin (BCG), provides variable protection against pulmonary TB, especially in adults. Hence, novel TB vaccine approaches are urgently needed. Both Th1 and Th17 responses are necessary for protection against TB, yet effective adjuvants and vaccine delivery systems for inducing robust Th1 and Th17 immunity are lacking. Herein we describe a synthetic Mincle agonist, UM-1098, and a silica nanoparticle delivery system that drives Th1/Th17 responses to *Mtb* antigens. Stimulation of human peripheral blood mononuclear cells (hPBMCs) with UM-1098 induced high levels of Th17 polarizing cytokines IL-6, IL-1β, IL-23 as well as IL-12p70, IL-4 and TNF-α in vitro. PBMCs from both C57BL/6 and BALB/c mice responded with a similar cytokine pattern in vitro and in vivo. Importantly, intramuscular (I.M.) vaccination with UM-1098-adjuvanted TB antigen M72 resulted in significantly higher antigen-specific IFN-γ and IL-17A levels in C57BL/6 wt mice than Mincle KO mice. Vaccination of C57BL/6 wt mice with immunodominant *Mtb* antigens ESAT6/Ag85B or M72 resulted in predominantly Th1 and Th17 responses and induced antigen-specific serum antibodies. Notably, in a virulent *Mtb* challenge model, vaccination with UM-1098 adjuvanted ESAT6/Ag85B or M72 significantly reduced lung bacterial burden when compared with unvaccinated mice and protection occurred in the absence of pulmonary inflammation. These data demonstrate that the synthetic Mincle agonist UM-1098 induces strong Th1 and Th17 immunity after vaccination with *Mtb* antigens and provides protection against *Mtb* infection in mice.

## Introduction

Tuberculosis (TB) is a major global health concern having caused 6.4 million new infections and 1.5 million deaths in 2021 alone^1^. Tuberculosis is the leading cause of death worldwide due to a single infectious agent, with the exception of SARS-CoV-2 in 2020-2021^1^. A quarter of the world’s population is considered latently infected with TB^2^ and according to the Centers of Disease Control and Prevention (CDC), 5-10% of latently infected individuals will likely progress to active TB during their lifetime if they do not receive treatment^3^. Antibiotic treatment of TB is not only lengthy and costly but becoming more challenging due to the emergence of multidrug-resistant (MDR) and extensive drug-resistant (XDR) *Mtb* strains^4,5^. In 2021, 3.6% of new TB infections and 18% of previously treated cases were caused by MDR and rifampicin-resistant *Mtb*^1^. Therefore, prevention of infection and disease progression is one of the main goals of the World Health Organization’s *End TB Strategy*^6^. Unfortunately, the only licensed vaccine against TB, Bacille Calmette-Guérin (BCG), has varying efficacy in preventing disease (0-80%) and does not effectively protect against pulmonary TB in adults^7–10^. Hence, there is an urgent need to develop new and more efficacious TB vaccines. Current vaccine candidates in clinical trials are based on whole cell killed or attenuated mycobacteria, viral vectors or adjuvanted recombinant proteins which have less potential to induce serious adverse events compared to live or attenuated bacteria^11,12^. A challenge in the TB vaccine development pipeline however is the lack of defined correlates of protective immunity^13,14^. A Th1 immune response characterized by strong interferon-gamma (IFN-γ) production is generally believed to be necessary^15–19^ but not sufficient for protection against TB^18,20–22^. More recently, a role for T helper type 17 (Th17) immune responses in protection against TB has been identified^22–25^. However, uncontrolled Interleukin (IL)-17 production and subsequent inflammation can be detrimental to the host and must be tightly regulated^26,27^. A balanced Th1 and Th17 immunity therefore appears to be ideal for protective immunity to TB^28–30^.

Adjuvants targeting specific pathogen recognition receptors (PRRs) can be powerful tools for skewing the immune response to vaccination in a desired direction^31^. In fact, adjuvants targeting Toll like receptors (TLRs) and other PRRs have been used in TB vaccine research to induce different types of Th cell responses^13,32^. Similar to TLRs, C-type lectin receptors (CLRs) can be targeted by adjuvants and their activation typically drives Th17 immune responses via SYK/CARD9/NF-κB signaling^33,34^. Macrophage-Inducible C-type Lectin (Mincle), expressed on myeloid cells, is a member of the CLR family recognizing glycolipids such as the mycobacterial cord factor trehalose-6,6-dimycolate (TDM)^35,36^. The synthetic TDM analogue trehalose-6,6-dibehenate (TDB) has been shown to induce Th17 responses in mice but not in humans^37–40^ and to date no Th17 polarizing adjuvant is approved for use in humans (NIH Vaccine Adjuvant Compendium: https://vac.niaid.nih.gov). The recent discovery of new Mincle ligands and extensive structure-activity investigations have identified novel ligands with remarkable activity in both human and murine systems^41–46^. One highly active compound, UM-1098, was chosen for advancement as an adjuvant for TB vaccination after formulation optimization and in vitro and in vivo compound screening and dose finding studies.

Past research has emphasized the importance of efficient codelivery of antigen and adjuvant to the same antigen presenting cell (APC), thereby enhancing antigen uptake and presentation^47^. Nanoparticles have gained significant attention in the field of vaccine development due to their potential for antigen and adjuvant co-delivery, rapid uptake by APCs, biodegradability and biocompatibility^48–51^. We recently described silica nanoparticles as a customizable co-delivery platform for CLR agonists and described the development of amine-functionalized silica nanoparticles (A-SNPs) as a successful co-delivery platform for Mincle agonists and antigen^52^.

The primary objective of the current study was to contribute to the field of TB vaccine research by testing the immunogenicity and efficacy of a synthetic Mincle adjuvant and vaccine delivery system primarily driving Th17 type immune responses to benchmark recombinant antigens for TB.

**Fig. 1:**
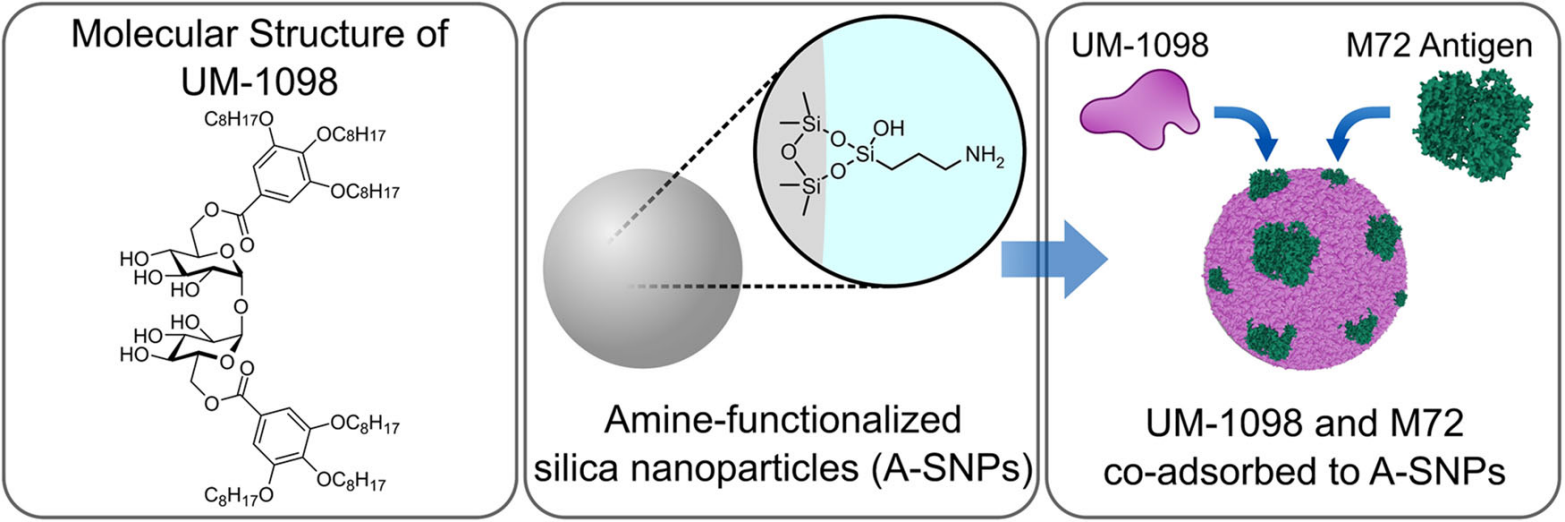
Schematic representing the Mincle agonist UM-1098 and its adsorption to Amine-functionalized silica nanoparticles. The first panel shows the chemical structure of UM-1098. The second panel shows amine-functionalized silica nanoparticles (A-SNPs). The third panel shows UM-1098 and the antigen M72 co-adsorbed to A-SNPs. Image was generated using Inkscape 1.2.2, representative protein structure was from RCBS.org (PDB ID: 1OVA)^98,99^.

Figure 1 shows the chemical structure of UM-1098 and a graphical representation of its co-adsorption to A-SNPs with the *Mtb* antigen M72^53^.

## Results

### UM-1098 induces high levels of IL-6, IL-1β, IL-23 and TNF-α in human PBMCs

**Fig. 2:**
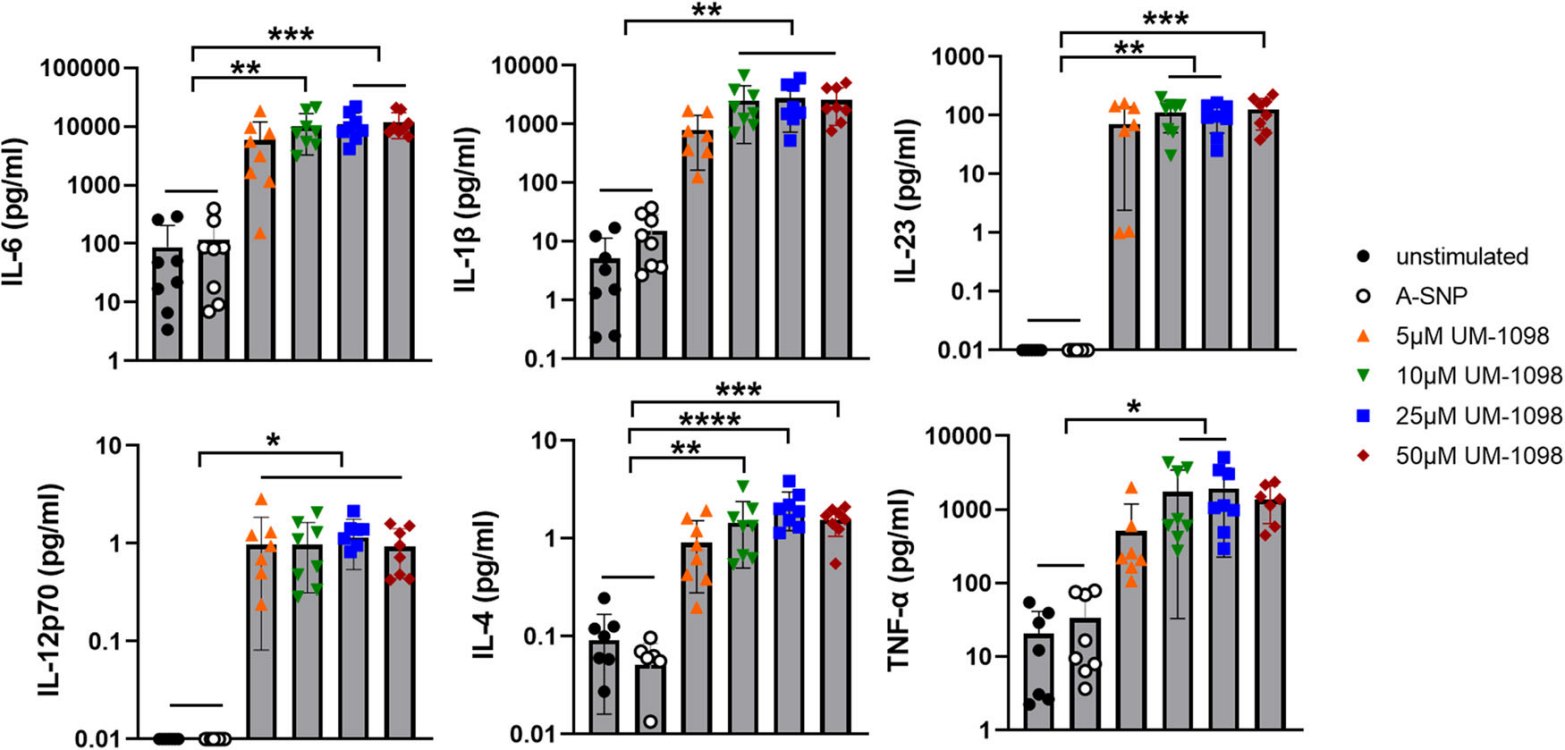
Innate cytokine profile of human PBMCs stimulated with UM-1098/A-SNP. Human PBMCs (*n* = 8 donors) were stimulated in vitro for 24 h with indicated concentrations of UM-1098. Unstimulated cells and PBMCs incubated with blank A-SNPs volume matched to 50 µM UM-1098 served as controls. Cytokine levels in supernatants were measured using MesoScale Discovery (MSD) U-PLEX Assay. Data was analyzed by Ordinary one-way ANOVA with Tukey’s multiple comparisons test and is represented as the mean and SD on a log scale. Significances were considered as follows: *p < 0.05, **p < 0.01, ***p < 0.001, ****p < 0.0001.

As a first step in understanding the immune profile of UM-1098, the ability of UM-1098 adsorbed to 200 nm A-SNPs (Fig. 1) to induce various T-cell polarizing innate cytokines in hPBMCs (in the absence of a protein antigen) was evaluated. Stimulation of hPBMCs with 10-50 µM UM-1098/A-SNP induced significantly higher levels of the Th17 polarizing cytokines IL-6, IL-1β, and IL-23 than stimulation with blank A-SNPs or mock stimulation with media (Fig. 2, top row, *p* < 0.01 and *p* < 0.001). Additionally, PBMCs stimulated with 10 or 25 µM UM-1098 responded with significantly higher TNF-α levels than unstimulated or blank A-SNP stimulated PBMCs (Fig. 2, bottom right, *p* < 0.05). Interestingly, UM-1098 also induced significantly higher levels of IL-12p70 and IL-4 in hPBMCs than blank A-SNPs or mock stimulation although absolute IL-12p70 and IL-4 levels were noticeably lower than Th17 polarizing cytokines (Fig. 2, bottom left and center, *p* < 0.05 for IL-12p70, *p* < 0.01, *p* < 0.001 and *p* < 0.0001 for IL-4). In conclusion, UM-1098 stimulation induced significant secretion of all three Th17 polarizing cytokines IL-6, IL-1β, IL-23 as well as TNF-α and low but significant levels of IL-12p70 and IL-4 in hPBMCs.

After having determined the innate cytokine profile of hPBMCs in response to UM-1098/A-SNP, the innate immune profile was compared to the Mincle targeting adjuvant formulation CAF01. CAF01 is made up of the Mincle agonist TDB formulated in liposomes containing the quaternary ammonium compound dimethyldioctadecylammonium (DDA). Both TDB and DDA are known to have immunostimulatory properties contributing to the adjuvant activity of CAF01 in mice^54^ and humans^39^.

**Fig. 3:**
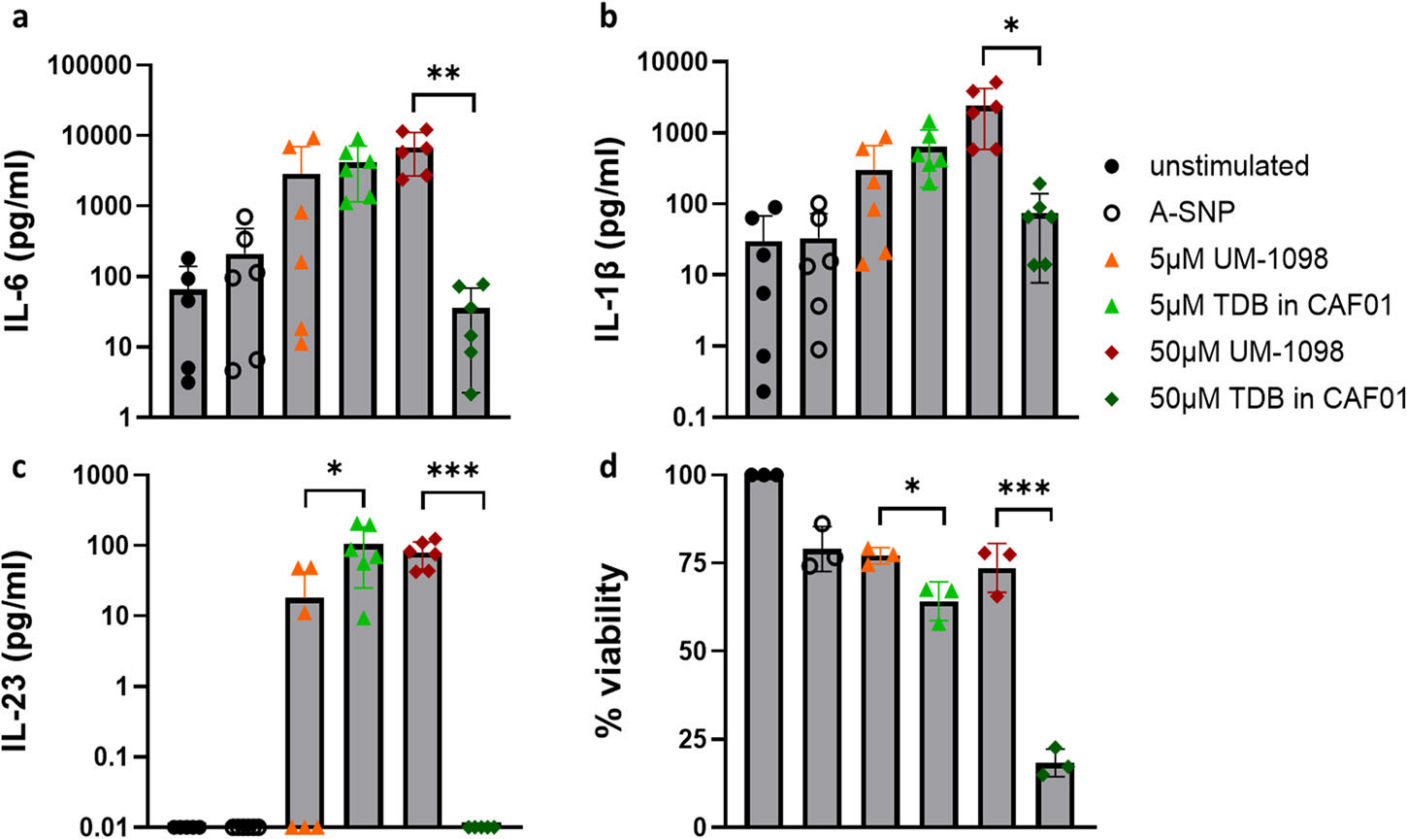
Th17 polarizing innate cytokine profile and viability of human PBMCs stimulated with UM-1098/A-SNP or CAF01. Human PBMCs (*n* = 6 donors) were stimulated in vitro for 24 h with indicated concentrations of UM-1098 or CAF01. Unstimulated cells and PBMCs incubated with blank A-SNPs volume matched to 50 µM UM-1098 served as controls. IL-6 (**a**), IL-1β (**b**), IL-23 (**c**) levels were measured in supernatants using MesoScale Discovery (MSD) U-PLEX Assay. Viability was assessed using CellTiter-Glo® Luminescent Cell Viability Assay (Promega) for cells from three donors and is shown as % of unstimulated cells (**d**). Data was analyzed by two unpaired two-tailed t tests comparing dose matched amounts of UM-1098 and CAF01. Data is represented as the mean and SD on a log scale (**a**–**c**) or linear scale (**d**). Significances were considered as follows: *p < 0.05, ** *p* < 0.01, ***p < 0.001, **** *p* < 0.0001.

Evaluation of secreted Th17 polarizing innate cytokines revealed that 5 µM UM-1098 induced similar levels of IL-6 and IL-1β in hPBMCs as 5 µM TDB formulated in CAF01 (Fig. 3a, b). Levels of IL-23 were significantly higher in hPBMCs stimulated with 5 µM CAF01 compared to 5 µM UM-1098 (Fig. 3c, *p* < 0.05). In contrast, 50 µM UM-1098 induced significantly higher levels of IL-6, IL-1β and IL-23 in hPBMCs than 50 µM TDB in CAF01 (Fig. 3a–c, *p* < 0.001, *p* < 0.01, *p* < 0.05). Concomitantly, cells stimulated with 5 or 50 µM CAF01 had significantly reduced viability compared to cells stimulated with equimolar amounts of UM-1098 (Fig. 3d, *p* < 0.05 and *p* < 0.001). Taken together, at a 5 µM dose, UM-1098 induced similar levels of Th17 polarizing innate cytokines as CAF01 whereas 50 µM UM-1098 induced significantly greater cytokine levels and significantly less cytotoxicity than equimolar amounts of TDB formulated in CAF01.

### UM-1098 induces Th17, Th1, Th2 polarizing innate cytokines in mouse PBMCs

**Fig. 4:**
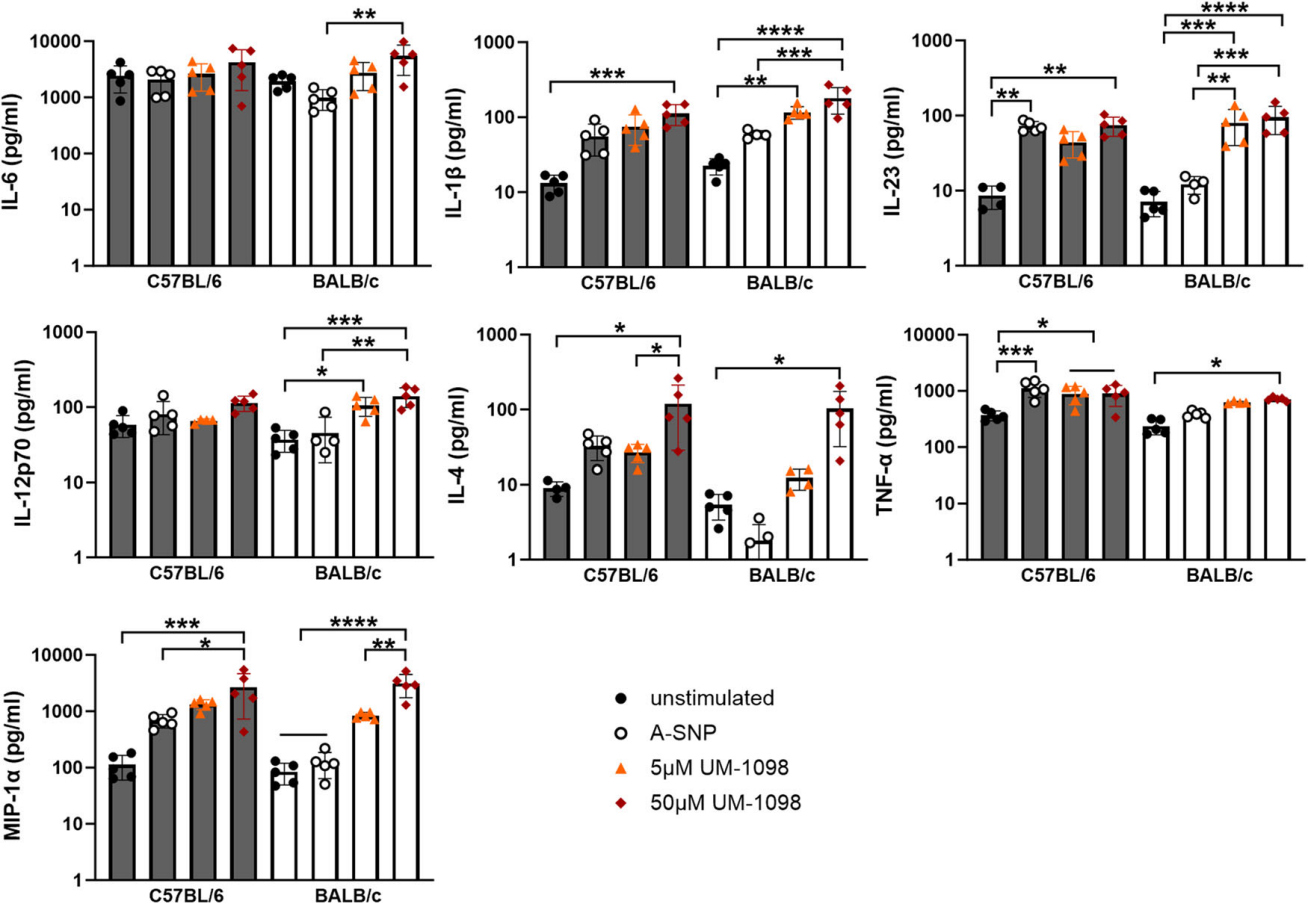
Innate cytokine profile of murine PBMCs stimulated with UM-1098/A-SNP. PBMCs from C57BL/6 and BALB/c mice were stimulated in vitro for 24 h with indicated concentrations of UM-1098. Unstimulated cells and PBMCs incubated with blank A-SNPs volume matched to 50 µM UM-1098 served as controls. Cytokine levels in supernatants were measured using MesoScale Discovery (MSD) U-PLEX Assay. Data is represented on a log scale and shown as the mean and SD of 5 experimental replicates performed with pooled blood of the respective mouse strains. Data was analyzed by Ordinary one-way ANOVA with Tukey’s multiple comparisons test. Significances were considered as follows: Significances were considered as follows: *p < 0.05, ** *p* < 0.01, ***p < 0.001, **** *p* < 0.0001.

Based on the results obtained for human PBMCs and as a screening method prior to using UM-1098 as an adjuvant in vivo, we investigated the innate cytokine profile of mouse PBMCs after UM-1098/A-SNP stimulation. C57BL/6 mice tend to mount a Th1 biased immune response and BALB/c mice a Th2 biased immune response^55,56^, but little is known about the Th17 biased response of these two commonly used mouse strains. We therefore compared PBMCs from both mouse strains (Fig. 4).

Noteworthily, UM-1098 induced significantly higher levels of the Th17 polarizing cytokines IL-1β and IL-23 as well as TNF-α and IL-4 in PBMCs from both strains compared to unstimulated controls (Fig. 4, *p* < 0.01, *p* < 0.001, *p* < 0.0001 for IL-1β and IL-23, *p* < 0.05 for IL-4 and TNF-α). Additionally, PBMCs from both mouse strains secreted more IL-6 and IL-12p70 in response to UM-1098 than mock stimulation but the difference was only significant for BALB/c mice (*p* < 0.01). IL-6 was indeed the cytokine released in the highest absolute amounts with 50 µM UM-1098 inducing over 5000 pg/ml in PBMCs from both strains (Fig. 4 first panel). However, baseline levels of IL-6 were high in PBMCs from both strains treated with media alone (1943 and 2423 pg/ml IL-6 for BALB/c and C57BL/6 respectively). MIP-1α was included in the cytokine panel because several studies have suggested this cytokine contributed to polarization of T_0_ cells toward a Th1 phenotype^57,58^. UM-1098 used at 50 µM induced significant levels of MIP-1α in PBMCs from both mouse strains compared to controls (Fig. 4, third row, *p* < 0.05, *p* < 0.001, *p* < 0.0001). Interestingly, there were no significant differences between BALB/c and C57BL/6 PBMCs stimulated with 5 or 50 µM UM-1098 for any of the investigated cytokines at the 24 h timepoint.

To summarize, PBMCs from both C57BL/6 and BALB/c mice responded to stimulation with 50 µM UM-1098 with a similar cytokine pattern (IL-1β, IL-23, TNF-α, MIP-1α, IL-4) as human PBMCs albeit absolute values differed between species (compare Figs. 2, 3, 4).

### UM-1098 induces Th17 and Th1 polarizing innate cytokines in mice following I.V. administration

**Fig. 5:**
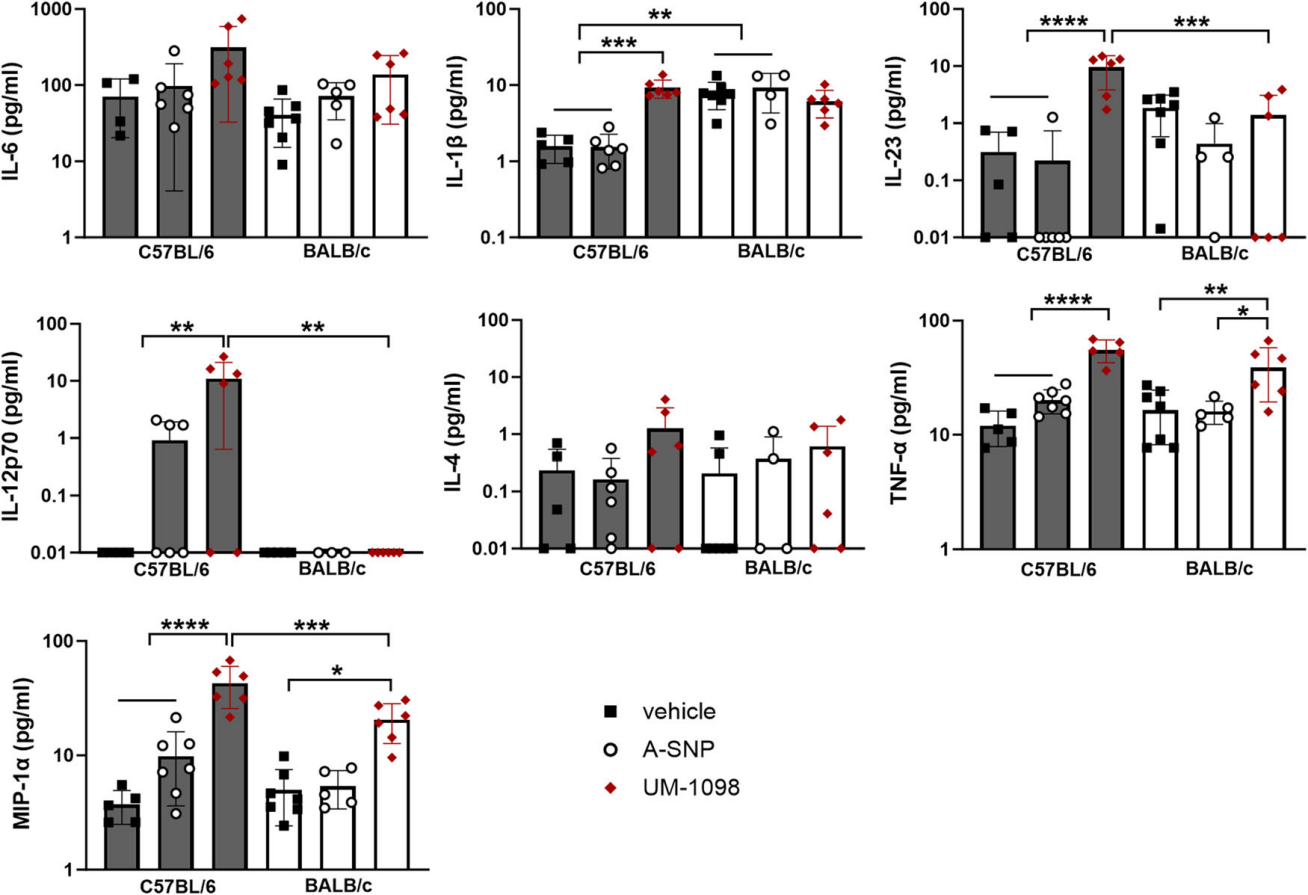
Innate serum cytokine profile of mice 4 h after intravenous administration of UM-1098/A-SNP. C57BL/6 and BALB/c wt mice (*n* = 7 mice/group) were injected I.V. with 250 nmol UM-1098/A-SNP, volume matched blank A-SNPs or vehicle (2% glycerol). Innate serum cytokines were measured in serum 4 h post injection using the MesoScale Discovery (MSD) U-PLEX Assay. Data is represented on a log scale with each data point representing one animal. Data was analyzed by Ordinary one-way ANOVA with Tukey’s multiple comparisons test and is represented as the mean and SD. Significances were considered as follows: *p < 0.05, **p < 0.01, ***p < 0.001, ****p < 0.0001.

**Fig. 6:**
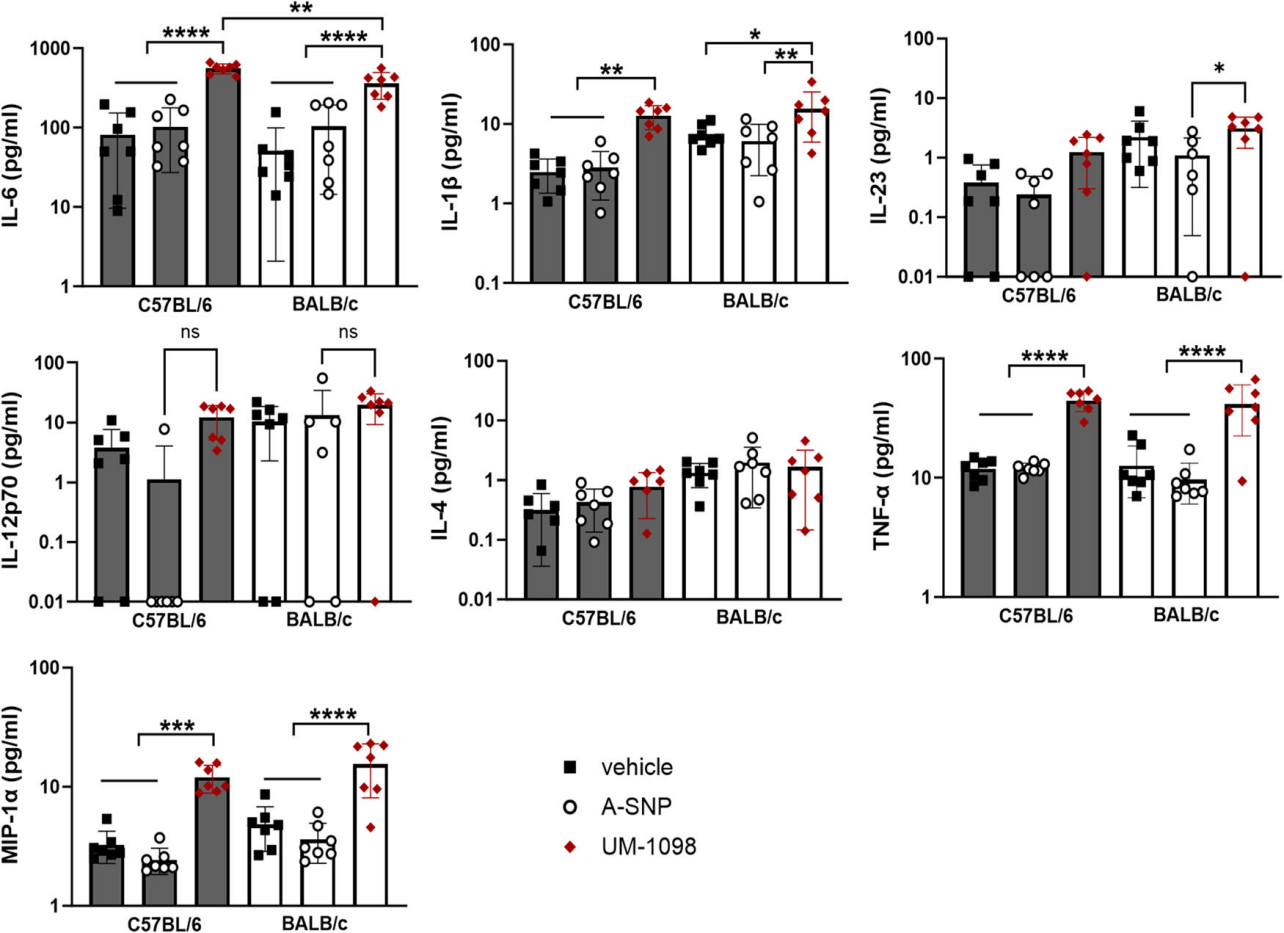
Innate serum cytokine profile of mice 24 h after intravenous administration of UM-1098/A-SNP. C57BL/6 and BALB/c wt mice (*n* = 7 mice/group) were injected I.V. with 250 nmol UM-1098/A-SNP, volume matched blank A-SNPs or vehicle (2% glycerol). Innate serum cytokines were measured in serum 24 h post injection using the MesoScale Discovery (MSD) U-PLEX Assay. Data is represented on a log scale with each data point representing one animal. Data was analyzed by Ordinary one-way ANOVA with Tukey’s multiple comparisons test and is represented as the mean and SD. Significances were considered as follows: *p < 0.05, **p < 0.01, ***p < 0.001, ****p < 0.0001.

In order to confirm the cytokine pattern seen after in vitro stimulation of PBMCs and to assess the safety of systemic administration of UM-1098, mice were injected intravenously (I.V.) with UM-1098/A-SNP and serum cytokines were analyzed at 4 h (Fig. 5) and 24 h (Fig. 6) post injection. Mice did not show overt signs of systemic toxicity at any time after I.V. administration of UM-1098/A-SNP. With regard to innate cytokines in serum, UM-1098 induced Th17 and Th1 but no significant levels of Th2 polarizing cytokines after I.V. injection in both C57BL/6 and BALB/c mice (Figs. 5, 6). Serum levels of IL-6, IL-1β, IL-23, TNF-α, MIP-1α were significantly higher in C57BL/6 and BALB/c mice injected with UM-1098 than mice injected with vehicle or blank A-SNPs at 4 and/or 24 h (Figs. 5, 6, *p* < 0.05, *p* < 0.01, *p* < 0.001, *p* < 0.0001). Interestingly and unlike mouse PBMCs, we measured significant strain differences with regard to serum levels of IL-6, IL-23, IL-12p70 and MIP-1α: C57BL/6 mice had significantly higher IL-23 (*p* < 0.001), IL-12p70 (*p* < 0.01), MIP-1α (*p* < 0.001) levels in serum 4 h post UM-1098 injections (Fig. 5). Additionally, C57BL/6 mice had significantly higher IL-6 serum levels than BALB/c mice 24 h after I.V. injection with UM-1098 (Fig. 6, first panel, *p* < 0.01). Lastly, levels of the Th2 polarizing cytokine IL-4 were low in all mice at both investigated timepoints and no significant difference was found between mice injected with UM-1098 or controls.

Taken together, I.V. injection of UM-1098 resulted in a similar pattern of Th17 and Th1 polarizing innate cytokines as in vitro stimulation of mouse and human PBMCs. However, absolute cytokine amounts were lower in vivo than in vitro and no significant induction of IL-4 could be measured in vivo (Figs. 4–6). Importantly, cytokine kinetics differed between strains with C57BL/6 mice responding to I.V. injection of UM-1098 with significantly higher IL-23, IL-12p70 and MIP-1α levels than BALB/c mice at 4 h but not at 24 h when cytokine levels were similar between strains except significantly higher IL-6 in C57BL/6 mice.

### I.M vaccination with M72 + UM-1098 induces a Th1/Th17 response in C57BL/6 but not in Mincle KO mice

As PBMC data revealed induction of Th1 and Th17 polarizing innate cytokines by UM-1098 in two genetically diverse mouse strains, the adjuvanticity of UM-1098 was evaluated in vivo next. For these studies UM-1098 was combined with the *Mtb* fusion protein M72^59^, as this antigen has been successfully used in human TB vaccine trials^59^. The aim of this first pilot in vivo study was to determine if UM-1098-mediated adjuvant activity was dependent on signaling through Mincle through the use of Mincle KO mice and the corresponding C57BL/6 wt strain. Additionally, potential differences in the immune responses to vaccination between C57BL/6 and BALB/c mice were investigated to determine if the different innate cytokine kinetics noted above were predictive of immunogenicity outcomes following vaccination. Mice were vaccinated I.M. three times and Th cell responses were analyzed in spleens and draining lymph nodes 21 days post tertiary injection. Vaccine induced immune responses were defined as Th1 if significantly greater levels of IFN-γ were present after vaccination and antigen re-stimulation compared to naïve controls. Analogously, immune responses were defined as Th17 if significantly greater levels of IL-17A were present after vaccination and antigen re-stimulation compared to naïve controls. If both IFN-γ and IL-17A were measured, the immune response was defined as “Th1/Th17” or “Th1 and Th17”. Of note, some mice (less than 50%) vaccinated with M72 + UM-1098 showed signs of local reactogenicity in the form of limping 24 h post vaccination that resolved within 48 h. No signs of systemic reactogenicity was observed in mice vaccinated with M72 + UM-1098 or respective controls. This finding will be carefully followed in future small and large animal studies to assess possible injection site reactions with this adjuvant formulation.

**Fig. 7:**
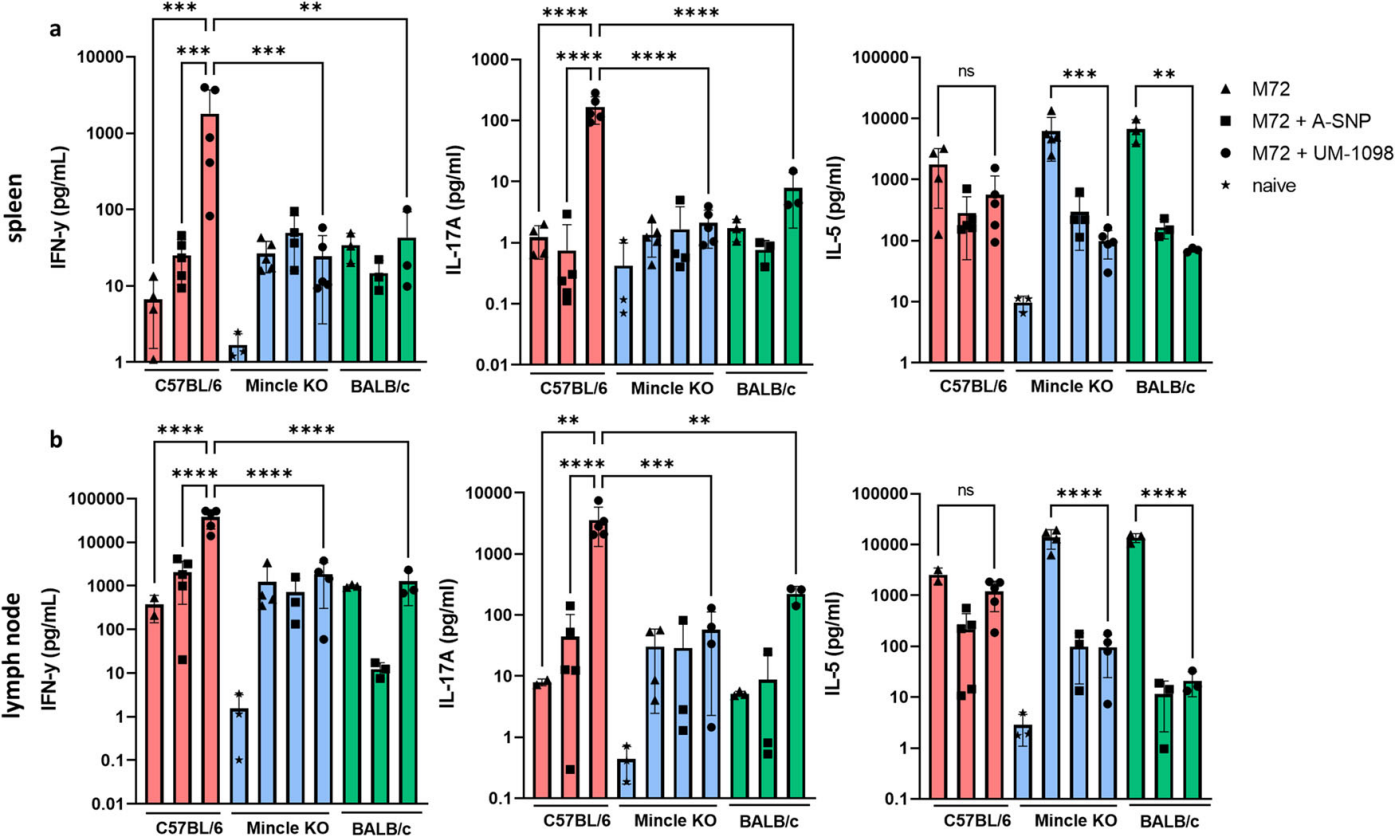
Vaccination with UM-1098 adjuvanted M72 induces significantly greater Th1 and Th17 responses in C57BL/5 than Mincle KO and BALB/c mice. C57BL/6 wt (red bars), Mincle KO (blue bars) and BALB/c wt (green bars) mice (*n* = 3–5 animals/group) were vaccinated three times with either M72 alone, M72 + blank A-SNPs or M72 + UM-1098/A-SNP. Unvaccinated Mincle KO mice served as naïve controls. Mice were sacrificed three weeks post tertiary injection and single cell suspensions of splenocytes (**a**) and draining lymph nodes (**b**) were re-stimulated with antigen for 72 h. Cytokines were measured in supernatants using the MesoScale Discovery (MSD) U-PLEX Assay. Data is represented on a log scale with each data point representing one animal. Data was analyzed by Ordinary one-way ANOVA with Tukey’s multiple comparisons test and is represented as the mean and SD. Significances were considered as follows: *p < 0.05, **p < 0.01, ***p < 0.001, ****p < 0.0001.

C57BL/6 wt mice vaccinated with M72 + UM-1098 had significantly higher IFN-γ (*p* < 0.001 and *p* < 0.0001) and IL-17A (*p* < 0.01 and *p* < 0.0001) concentrations in the spleens (Fig. 7a) and draining lymph nodes (Fig. 7b) after antigen re-stimulation than C57BL/6 mice vaccinated with M72 alone or M72 + blank A-SNPs. Importantly, IFN-γ and IL-17A were also significantly higher in the lymph nodes and spleens of C57BL/6 wt mice than Mincle KO (*p* < 0.001 and *p* < 0.0001) and BALB/c (*p* < 0.01 and *p* < 0.0001) mice after vaccination with M72 + UM-1098 (Fig. 7). In BALB/c mice, IL-17 responses were greater after vaccination with M72 + UM-1098 than vaccination with M72 alone or M72 + A-SNP (blank) but the difference was not significant (Fig. 7). IL-5 levels in the draining lymph nodes and spleens were not significantly different in C57BL/6 wt, Mincle KO or BALB/c mice vaccinated with M72 + UM-1098 (Fig. 7). However, in Mincle KO and BALB/c mice, vaccination with M72 + UM-1098 resulted in significantly lower IL-5 levels in draining lymph nodes and spleens than vaccination with antigen alone (Fig. 7*p* < 0.01 and *p* < 0.001 for spleens, *p* < 0.0001 for lymph nodes).

Taken together, data from a pilot study indicate that vaccination of C57BL/6 wt mice with M72 + UM-1098 results in significantly higher Th1 and Th17 responses in C57BL/6 wt but not in Mincle KO or BALB/c mice. These data suggest that UM-1098 is a Mincle-specific agonist and that C57BL/6 mice exhibit a stronger antigen-specific Th1/Th17 response than BALB/c mice, after vaccination with M72 + UM-1098.

### Vaccination of C57BL/6 wt mice with M72 + UM-1098 induces antigen specific IgG and IgG1

**Fig. 8:**
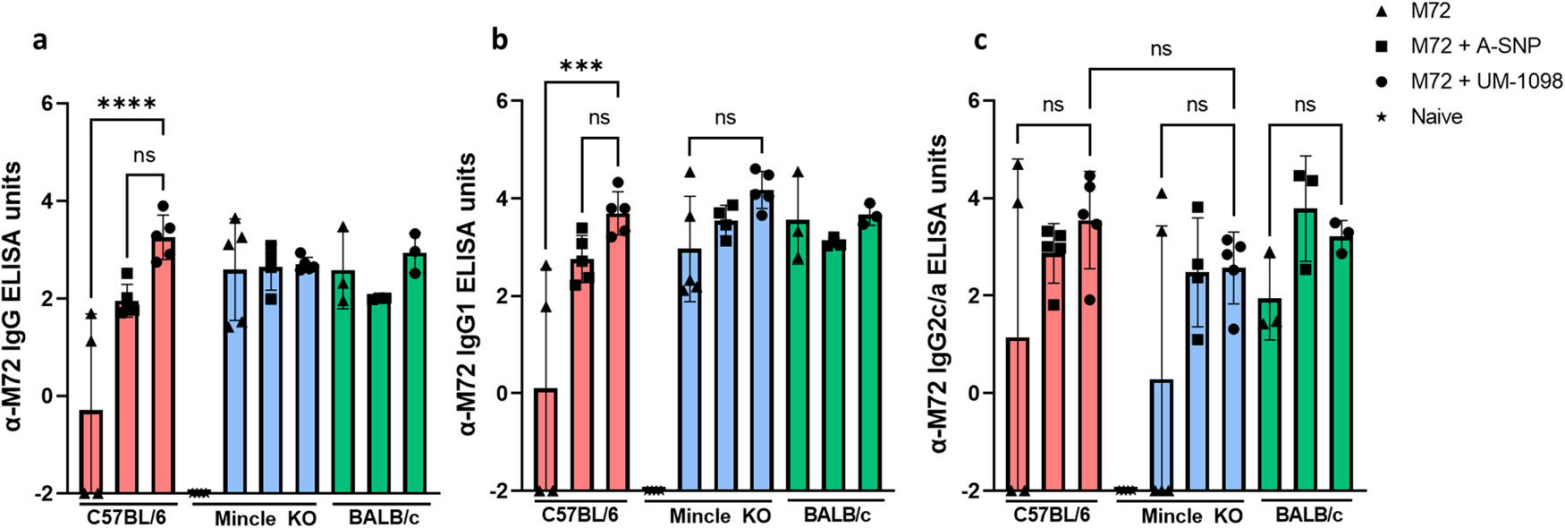
Vaccination with UM-1098 adjuvanted M72 induces antigen specific IgG and IgG1 in C57BL/6 wt mice. C57BL/6 wt (red bars), Mincle KO (blue bars) and BALB/c (green bars) wt mice (*n* = 3–5 animals/group) were vaccinated three times with either M72 alone, M72 + blank A-SNPs or M72 + UM-1098/A-SNP. Unvaccinated Mincle KO mice served as naïve controls. Mice were sacrificed three weeks post tertiary injection and antigen specific IgG (**a**), IgG1 (**b**), IgG2c/a (**c**) was measured in serum via ELISA. Data was log transformed and is represented on a linear scale with each data point representing one animal. Data was analyzed by Ordinary one-way ANOVA with Tukey’s multiple comparisons test and shown as the mean and SD. Significances were considered as follows: *p < 0.05, ** *p* < 0.01, ***p < 0.001, ****p < 0.0001.

In addition to measuring Th cell responses, humoral immune response to vaccination with M72 + UM-1098 were evaluated (Fig. 8). Analyzing antigen-specific antibody in serum 21d post tertiary vaccination, C57BL/6 wt mice vaccinated with M72 + UM-1098 had significantly higher levels of M72 specific IgG and IgG1 than C57BL/6 wt mice vaccinated with M72 alone (Fig. 8a, b; *p* < 0.001). In contrast, no significant differences were identified between M72 alone and M72 + UM-1098 vaccinated Mincle KO or BALB/c mice. With regard to Th1 associated IgG2, C57BL/6 mice vaccinated with M72 + UM-1098 had higher antigen-specific antibody levels in serum than wt mice vaccinated with M72 alone or Mincle KO mice vaccinated with M72 + UM-1098, but the differences were not significant (Fig. 8c). Additionally, no significant differences were found between C57BL/6 and BALB/c mice for either IgG, IgG2 or IgG1. In summary, antibody data indicate that adjuvanting M72 with UM-1098 leads to a significant boost in antigen specific serum IgG and IgG1 in C57BL/6 wt but not in Mincle KO or BALB/c mice.

### I.M vaccination of C57BL/6 mice with ESAT6/Ag85B + UM-1098 induces mixed T cell responses in vivo

As noted above, UM-1098 and the clinical candidate TB antigen M72 showed promising Th1/Th17 responses in vivo. To determine if the Th1/17 immunity induced by UM-1098 extended to other immunodominant TB antigens, adjuvanticity of UM-1098 in combination with TB antigens ESAT6 and Ag85B^60^ was evaluated. M72 was included in the second study as a clinical benchmark and to verify our previous results. As the combination of M72 + UM-1098 revealed superior adjuvanticity and Th1/17 polarization in C57BL/6 compared to BALB/c mice, further immunogenicity and *Mtb* challenge studies were performed in C57BL/6 mice only. As noted above, some mice (less than 50%) in the antigen + UM-1098 vaccinated group exhibited local reactogenicity in the form of limping 24 h post vaccination that resolved within 48 h. No signs of systemic reactogenicity were observed in mice vaccinated with antigen + UM-1098 or respective controls.

**Fig. 9:**
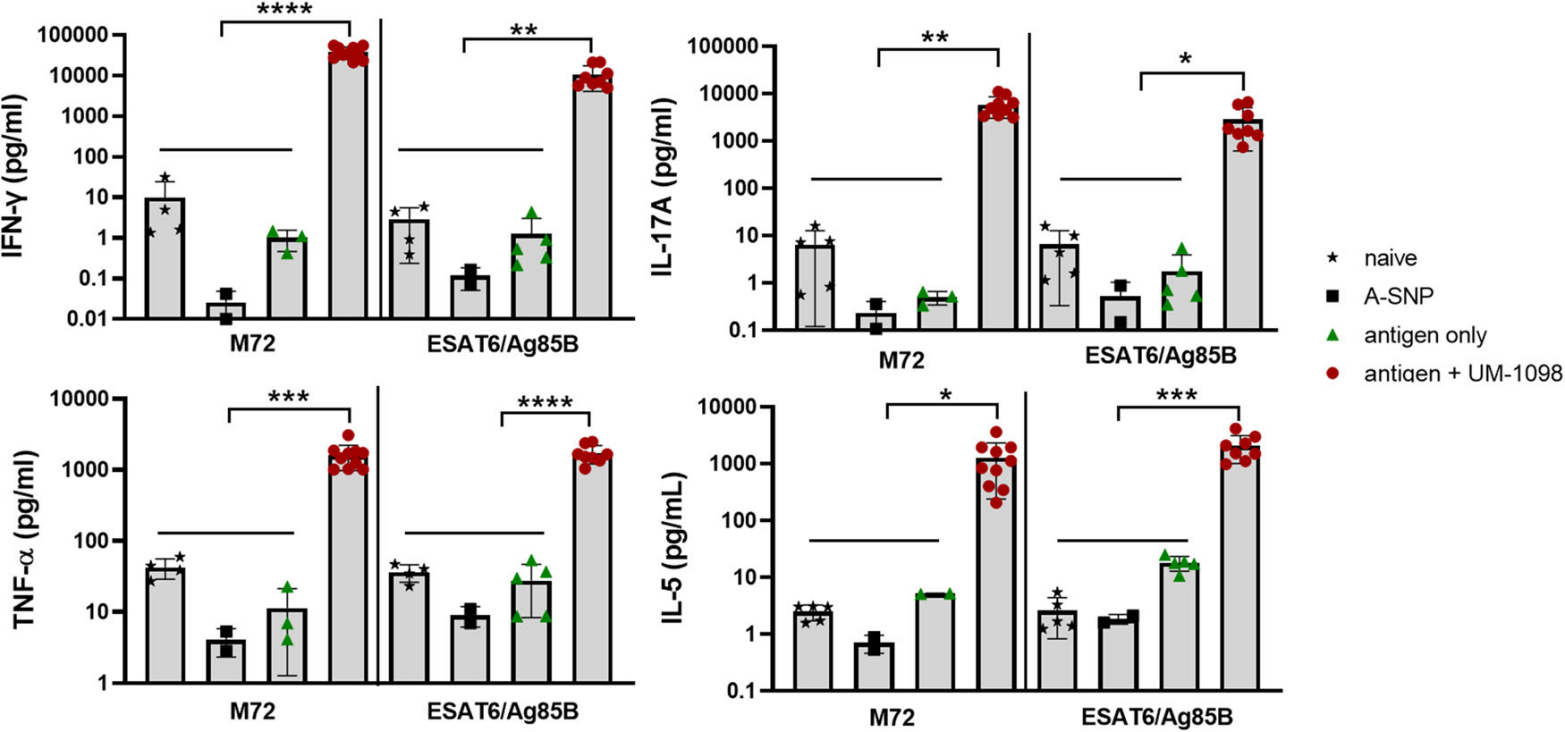
Vaccination with UM-1098 adjuvanted M72 or ESAT6/Ag85B induces mixed Th cell responses in C57BL/5 wt mice. C57BL/6 wt mice (*n* = 10 mice/group) were vaccinated three times I.M. with either blank A-SNPs, antigen alone, or antigen + UM-1098/A-SNP. Antigens were either M72 or the combination of ESAT6 and Ag85B. Unvaccinated mice served as naïve controls. Mice were sacrificed three weeks post tertiary injection and single cell suspensions of draining lymph nodes were re-stimulated with antigen for 72 h. Cells from mice immunized with a M72-containing vaccine were re-stimulated with M72 only whereas cells from mice immunized with an ESAT6/Ag85B-containing vaccine were re-stimulated with ESAT6/Ag85B. Cells from control mice (naïve or A-SNP vaccinated) were incubated with both antigens separately. Cytokines were measured in supernatants using the MesoScale Discovery (MSD) U-PLEX Assay. Data was analyzed by Ordinary one-way ANOVA with Tukey’s multiple comparisons test and is represented as the mean and SD on a log scale. Significances were considered as follows: *p < 0.05, ** *p* < 0.01, ***p < 0.001, ****p < 0.0001.

Vaccination with either M72 + UM-1098 or ESAT6/Ag85B + UM-1098 lead to significantly higher IFN-γ, IL-17A, TNF-α, IL-5 levels in the draining lymph nodes post antigen re-stimulation than vaccination with antigen alone or blank A-SNPs (Fig. 9, *p* < 0.05, *p* < 0.01, *p* < 0.001, *p* < 0.0001). Of note, the magnitude of the cytokine response differed considerably with IFN-γ being the predominantly secreted cytokine in the draining lymph nodes of antigen + UM-1098 vaccinated mice (Fig. 9 and Supplementary Fig. 3). IFN-γ concentrations in draining lymph nodes of antigen + UM-1098 vaccinated mice were significantly higher than IL-17A, TNF-α and IL-5 levels (Supplementary Fig. 3, *p* < 0.0001 for M72, *p* < 0.001 for ESAT6/Ag85B). Mice vaccinated with M72 + UM-1098 or ESAT6/Ag85B + UM-1098 secreted cytokines in the following order of magnitude IFN-γ > IL-17A > IL-5 indicating induction of Th1 > Th17 > Th2. Secreted cytokines in the spleens also revealed significant induction of IFN-γ, IL-17A, TNF-α and IL-5 in mice vaccinated with M72 or ESAT6/Ag85B + UM-1098 (Supplementary Fig. 4, *p* < 0.05, *p* < 0.01, *p* < 0.0001). Of note, M72 alone induced higher IL-5 levels in the first study (Fig. 7a) than the second study (Fig. 9) resulting in a significant increase in the antigen + UM-1098 vaccinated mice in this study only. Flow cytometric analysis of intracellular cytokines in the spleens confirmed secreted cytokine data (Supplementary Fig. 6): The percentages of CD4+ lymphocytes positive for IFN-γ, IL-17A, TNF-α, IL-5 and IL-2 were significantly higher in antigen + UM-1098 vaccinated mice compared to control groups (i.e. naïve, antigen only, A-SNP only vaccinated mice; *p* values ranging from *p* < 0.05 to *p* < 0.0001 depending on cytokine). Taken together, these data indicate that UM-1098 induces mixed Th1/17 cell responses in mice with a dominance of Th1 type responses in the draining lymph nodes.

### Vaccination of C57BL/6 wt mice with ESAT6/Ag85B + UM-1098 induces antigen specific IgG, IgG2c, IgG1

**Fig. 10:**
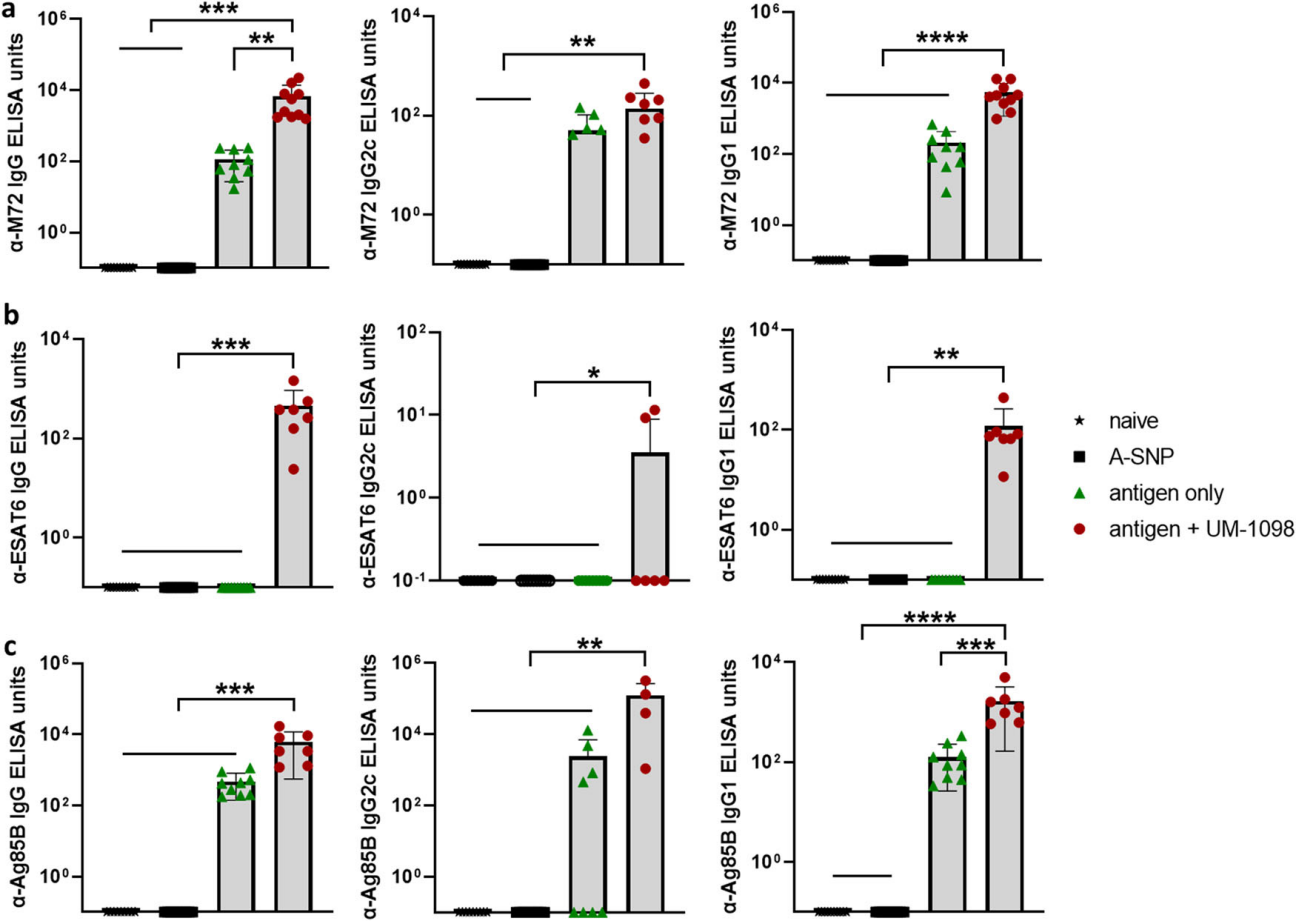
Vaccination with UM-1098 adjuvanted M72 or ESAT6/Ag85B induces antigen specific antibodies in C57BL/5 wt mice. Antigen specific IgG, IgG2c, IgG1 was measured by ELISA in serum of C57BL/6 wt mice (*n* = 10 mice/group) three weeks post tertiary vaccination with either blank A-SNPs, antigen alone, or antigen + UM-1098/A-SNP. Antigens were either M72 or a combination of ESAT6 and Ag85B. Unvaccinated mice served as naïve controls. M72-specific antibodies are shown in (**a**), ESAT6-specific antibodies in (**b**) and Ag85B-specific antibodies in (**c**). Data was analyzed by Ordinary one-way ANOVA with Tukey’s multiple comparisons test and is represented as the mean and SD on a log scale. Significances were considered as follows: *p < 0.05, ** *p* < 0.01, ***p < 0.001, **** *p* < 0.0001.

To complement the cell mediated immunity data, the antigen-specific antibody response was evaluated in sera of mice 21 days post tertiary vaccination. Mice vaccinated with M72 + UM-1098 had significantly higher antigen-specific serum IgG and IgG1 than naive mice or animals vaccinated with antigen or A-SNPs alone (Fig. 10a, *p* < 0.01, *p* < 0.001, *p* < 0.0001). M72-specific IgG2c was not significantly higher in mice vaccinated with M72 + UM-1098 in comparison to mice vaccinated with antigen alone. With regard to ESAT6/Ag85B, mice vaccinated with both antigens and UM-1098 had significantly higher ESAT6- and Ag85B-specific serum IgG, IgG2c, IgG1 than mice vaccinated with antigen alone, blank A-SNPs or naïve mice (Fig. 10b, c; *p* values ranging from *p* < 0.05 to *p* < 0.0001).

### Vaccination with UM-1098-adjuvanted TB antigens protects mice against *Mtb* challenge

**Fig. 11:**
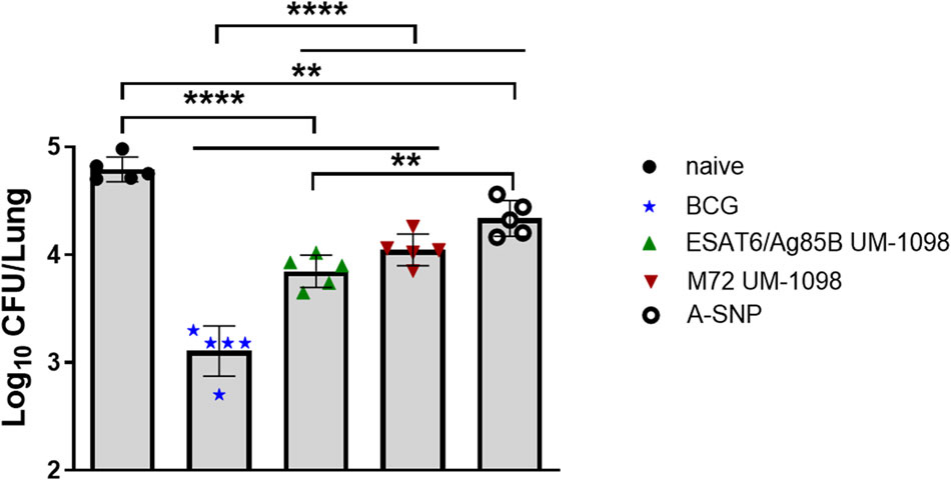
Vaccination with UM-1098 adjuvanted M72 or ESAT6/Ag85B reduces the number of *Mtb* CFUs in the lungs of C57BL/5 wt mice 30 d post infection. Five C57BL/6 mice per group were vaccinated I.M. with UM-1098/A-SNP adjuvanted M72 or ESAT6/Ag85B or blank A-SNPs three times two weeks apart. Unvaccinated or BCG vaccinated mice served as controls. Bacterial burden in the lungs was determined 30 days post infection with *Mtb* HN878 via plate counting. Data was analyzed by Ordinary one-way ANOVA with Tukey’s multiple comparisons test and is represented as the mean and SD. Significances were considered as follows: *p < 0.05, **p < 0.01, ***p < 0.001, ****p < 0.0001.

Since Th1/17 cell mediated immunity and humoral responses were induced in C57BL/6 mice vaccinated I.M. with UM-1098 and either M72 or ESAT6/Ag85B, protection against virulent *Mtb* challenge was evaluated. Mice were vaccinated three times, two weeks apart and challenged 30 days after the last booster (Supplementary Fig. 7). Vaccination of mice with UM-1098 in combination with either M72 or ESAT6/Ag85B significantly reduced lung bacterial burden compared to unvaccinated mice (Fig. 11, *p* < 0.0001). No significant difference was measured in lung CFUs between mice vaccinated with M72 + UM-1098 or ESAT6/AG85B + UM-1098. Interestingly, mice vaccinated with blank A-SNPs alone also had significantly less CFUs in the lungs than naïve mice (Fig. 11, *p* < 0.05). However, vaccination with either M72 + UM-1098 or ESAT6/Ag85B + UM-1098 resulted in lower bacterial burden in the lungs than vaccination with blank ASNPs and the difference was significant for the comparison with ESAT6/Ag85B + UM-1098 (Fig. 11, *p* < 0.05). Additionally, CFUs in the lungs were significantly lower in mice vaccinated with BCG than mice vaccinated with blank ASNPs or either of the two UM-1098 adjuvanted subunit vaccines (Fig. 11, *p* < 0.0001). Since Th17 responses as well as administration of BCG have the potential to cause lung inflammation^27,61^, the percentage of pulmonary inflammation post challenge was measured. While vaccination with either BCG, blank A-SNPs or UM-1098-adjuvanted TB antigens induced some lung inflammation, no significant differences were observed between the unvaccinated and vaccinated groups of mice 30d post *Mtb* challenge (Supplementary Figs. 8, 9).

To conclude, I.M. vaccination with M72 + UM-1098 or ESAT6/Ag85B + UM-1098 reduced bacterial burden in the lungs without causing increased pulmonary inflammation.

## Discussion

As TB remains a major global health concern, the development of a reliably effective TB vaccine is urgently needed. The importance of the CLR Mincle in protection against mycobacterial infection is ambiguous^62–66^. Some studies report Mincle KO mice to be more susceptible to mycobacterial infection than wt mice. For example, Behler et al. reported less IFN-γ producing T cells and higher mycobacterial burden in Mincle KO than wt mice after I.V. infection with BCG^63^. Similarly, Lee et al. found more CFUs in the lungs of Mincle KO than wt mice after challenge with *Mtb* strain Erdman^65^. In contrast, Heitmann et al. reported no difference in bacterial burden, granuloma formation or Th cell induction between wt and Mincle KO mice during active *Mtb* infection^66^. Lastly, Dubé et al. reported increased susceptibility to *Mtb* infection in Mincle KO mice at late stages of infection^64^. Despite these conflicting reports, Mincle represents a promising vaccine adjuvant target for TB due to its ability to drive Th1 and Th17 type immune responses, which are reported as necessary for protection against TB^30,67,68^. In the current study, the immune profile of the synthetic Mincle agonist UM-1098 and its potential as a Th1 and Th17 skewing adjuvant was investigated. UM-1098 induced mainly Th17 and Th1 polarizing innate cytokines in human and mouse PBMCs. Importantly, vaccination with different *Mtb* antigens co-adsorbed with UM-1098 onto A-SNPs led to predominantly Th1 and Th17 responses in mice. This finding is particularly significant as most adjuvants used in TB research favor Th1 immunity only^13,32^. Indeed, the Th1 and Th17 responses induced by vaccination with UM-1098 adjuvanted TB antigens coincided with protection against *Mtb* infection in a murine model.

A pilot study comparing Mincle KO and wt mice, suggested that UM-1098-mediated Th cell responses are Mincle-dependent as Mincle KO mice had significantly lower IFN-γ and IL-17A levels in spleens and draining lymph nodes than wt mice following vaccination with M72 + UM-1098. The adjuvant effect of UM-1098 on antibody production appeared to be independent of Mincle as both wt and Mincle KO mice had similar antigen-specific antibody titers in serum after vaccination with M72 + UM-1098. However, further studies are necessary to fully explore the mouse strain differences in relation to Mincle-mediated adaptive immunity.

Interestingly, C57BL/6 mice had higher levels of Th1 and Th17 polarizing innate cytokines in serum after I.V. injection of UM-1098 and mounted significantly greater Th1 and Th17 responses to vaccination with M72 + UM-1098 than BALB/c mice. We hypothesize that the difference in early serum cytokine kinetics seen after I.V. injection of UM-1098 contributed to the significantly higher antigen-specific IFN-γ and IL-17A levels seen in C57BL/6 compared to BALB/c mice after I.M. vaccination. As both C57BL/6 and BABL/c mice carry the Mincle gene (c*lec4e*), we speculate that unknown factors such as differences in Mincle expression or different splicing variants contribute to the divergent phenotype seen in C57BL/6 versus BALB/c mice. Even though the Th1/Th2 dichotomy of C57BL/6 and BALB/c is well known, fewer studies have investigated the differences in Th17 responses between those two strains. Albeit not using a vaccination approach, Ferreira et al. found significantly higher IL-17 levels in C57BL/6 than BALB/c mice after infection with *Trypanosoma cruzi*^69^. On the contrary, Garcia-Pelayo et al. showed significantly greater Th1 and Th17 responses in BALB/c mice than C57BL/6 mice after vaccination with BCG although no difference in bacterial burden was detected after challenge^70^. Similarly, Jiang et al. noted greater IL-23 release from BALB/c BMDCs than C57BL/6 cells after stimulation with *Chlamydia muridarum* and more IL-17 in BALB/c than C57BL/6 mice after infection with the same bacterium^71^. These divergent results in terms of Th17 responses of C57BL/6 and BALB/c mice could potentially be due to the very different models, pathogens and timepoints under investigation but future studies are needed to fully understand the differences in Th17 immunity between these two very commonly used mouse strains.

The concept of exploring Mincle as a Th17 inducing adjuvant target is not entirely new. The prominent Mincle agonist-based adjuvant CAF01 (a cationic liposomal formulation of TDB) has been used in TB vaccine research and found to induce Th1 and Th17 responses in mice. Several studies have reported that vaccination of mice with TB antigens and CAF01 induces long lasting Th1 and Th17 immunity and protection against *Mtb* challenge^40,54,72^. Unfortunately, despite promising Th17 inducing properties in mice, CAF01 did not induce significant Th17 responses in humans^39^. In vitro experiments comparing CAF01 to UM-1098 revealed that CAF01 had a dose dependent cytotoxic effect on hPBMCs which we did not observe with equimolar amounts of UM-1098 (Fig. 3). DDA is necessary for the stability of CAF01 and testing of DDA liposomes or TDB alone is not possible due to the low colloidal stability of these formulations. However, we attribute the cytotoxic effect observed with high doses of CAF01 to the presence of DDA rather than TDB because studies by Silva et al. and Inglut et al. have demonstrated concentration dependent cytotoxic effect of DDA-containing liposomes on macrophages and various cell lines^73,74^. This highlights that the choice of formulation for any adjuvant is paramount and careful characterization and in vitro screening should be performed prior to any in vivo study.

The immunogenicity of another Mincle targeting adjuvant was tested by Decout et al. who showed that the liposomal formulation of the synthetic TDB derivative GlcC14C18 induced IL-17 and IFN-γ in mice after intradermal vaccination with the *Mtb* antigen Ag85A and protected against virulent *Mtb* challenge^75^. Our results using the Mincle agonist UM-1098 to induce Th1 and Th17 responses in mice are therefore in line with the published literature on Mincle agonists. The strong Th17 polarizing cytokine response to UM-1098 seen in human PBMCs (Fig. 2) further supports advancement of this compound as a clinical adjuvant candidate for Th17 induction. A clinical TB vaccine candidate that has been found to induce Th17 responses in humans is MVA85A, an Ag85A expressing modified vaccinia Ankara virus not containing an adjuvant^76^. Boosting of BCG primed individuals with MVA85A induced antigen-specific Th1 and Th17 cells in infants, adolescents and adults^77–79^. However, the authors found Th17 responses to be lower in magnitude and to peak significantly later than Th1 responses and present a correlation between preexisting immunity to mycobacterial antigens and reduced capacity to produce IL-17A after immunization^79,80^. Additionally, MVA85A did not show protection against infection in humans despite the promising safety and immunogenicity profile^20^. Considering the proposed importance of Th17 responses for protection against TB, we hypothesize that the lack of protection against TB in humans after MVA85A vaccination may be related to insufficient or delayed Th17 responses, highlighting once again the need for a Th17 inducing adjuvant in humans.

Some current TB vaccine approaches explore the combination of BCG and a subunit vaccine, either administered together or sequentially. Coadministration of BCG and TB subunit vaccine H107/CAF01 consisting of eight *Mtb* antigens (including ESAT6) led to increased Th17 responses in mice and superior protection against *Mtb* than administration of BCG alone^81^. BCG has been reported to increase expression of Mincle on myeloid cells both in vitro and in vivo^82^. Despite the promising results in mice, when BCG was combined with a CAF01-adjuvanted subunit vaccine, Darrah et al. were unable to detect increased IL-17 production or improved protection when BCG-primed rhesus macaques were boosted I.M. with H56/CAF01^83^. This highlights once again the apparent difficultly to induce Th17 responses by vaccination in primates and underscores the need for and the potential benefit of an adjuvant that specifically induces Th17 or Th1/17 immunity in humans. The most successful adjuvanted subunit TB vaccine to date, M72/AS01_E_, consists of the TB antigen M72 and the adjuvant AS01_E_, a liposomal formulation of the TLR4 agonist monophosphoryl-lipid A (MPLA) and the saponin QS-21^53^. M72/AS01_E_ exhibited almost 50% reduction in progression to pulmonary TB in latently infected individuals as well as induction of polyfunctional CD4+ cells^59^. Yet, vaccination induced very low amounts of IL-17 + CD4+ cells in adults and adolescents^84–86^. This finding provides further evidence that Th1 responses alone provide partial protection and that improved TB vaccines need to elicit additional responses, such as Th17, to be more than 50% effective. In the current study, the ability of UM-1098 to induce antigen specific Th1 and Th17 responses in mice was demonstrated for multiple immunodominant TB antigens, but it remains unknown if those responses translate to humans. Although our study did not involve vaccinations of humans and data obtained from mice are not directly translatable to humans, our hPBMC data demonstrating high levels of Th17 polarizing innate cytokines after UM-1098 stimulation are promising and warrant future testing of UM-1098 as a Th1/Th17 skewing adjuvant in humans. However, Th1/Th17 immunity is likely not the only factor needed for protection against TB in humans and future studies should aim to confirm the importance of Th1/Th17 immunity in protection against TB in humans as well as identify additional correlates of protection against TB.

The ability of UM-1098 to induce Th1/Th17 responses in combination with different antigens highlights the versatility and potential of this adjuvant to be used in vaccines against any disease where Th17 and balanced Th1/Th17 is deemed necessary for vaccine-mediated protection. Examples of pathogens for which Th17 responses have been reported to be involved in protection and for which the utility of the UM-1098 adjuvant could be explored are *Bordetella pertussis*, *Aspergillus fumigatus*, *Candida albicans*, *Staphylococcus aureus, Mtb*, and *Klebsiella pneumoniae*^87–91^.

In summary, the synthetic Mincle agonist UM-1098 adsorbed to A-SNPs is a safe and promising Th1 and Th17 inducing adjuvant that provides protection against virulent *Mtb* challenge in mice when used in combination with different TB antigens.

## Methods

### Synthesis of UM-1098 and adsorption to A-SNPs

2,2’,3,3’,4,4’-Hexa-trimethylsilyl-α,α-D-trehalose (5.0 g, 6.4 mmol)^43^, was combined with commercially available 3,4,5-Tris(octyloxy)benzoic acid (7.2 mg, 14.2 mmol) and 4-(dimethylamino)pyridine (2.35 g, 19.2 mmol) as well as methylene chloride (250 mL) and cooled to 2–8 °C with an external ice bath. N,N’-diisopropylcarbodiimide methyl iodide (9.5 g, 32 mmol) was added and the stirred reaction was allowed to warm to ambient temperature and continued stirring for 60 h. The reaction was concentrated in vacuo and the crude residue was dissolved in methylene chloride (50 mL) and stirred with Trifluoroacetic acid (19.2 mL, 115 mmol) for 35 min. The resulting residue was subjected to column chromatography on silica gel (linear gradient from 0-30% methanol/ ethyl acetate). Fractions containing pure UM-1098 were combined, concentrated and dried under high vacuum yielding UM-1098 (5.24 g, 62%). ^1^H NMR (400 MHz, DMSO-d6) δ 7.13 (s, 4H), 5.17 (br, 2H), 4.98 (d, J = 8.9 Hz, 4H), 4.85 (d, J = 4.4 Hz, 2H), 4.46 (d, J = 9.2 Hz, 2H), 4.14 (br, 2H), 4.04 (br, 2H), 3.84 (br, 12H), 3.64 (br, 2H), 3.28 (br, 2H), 3.16 (br, 2H), 1.60 (br, 12H), 1.33 (br, 12H), 1.16 (br, 48H), 0.76 (br, 18H); ^13^C NMR (100 MHz, DMSO-d6) δ 165.17, 152.29, 141.39, 124.48, 107.13, 92.90, 72.71, 72.40, 71.67, 70.62, 69.70, 68.21, 64.48, 31.29, 31.22, 29.79, 28.88, 28.77, 28.76, 28.73, 25.59, 25.52, 22.08, 13.79, 13.76; HRMS (ESI + , m/z) calc (M + NH_4_): 1336.9237, found: 1336.9222. The reaction of UM-1098 synthesis is shown in Supplementary Fig. 1.

Methods for adsorption of UM-1098 to 10 mg/ml A-SNPs with a 200 nm diameter and characterization of adsorption were performed as previously described using agonists similar to UM-1098^52^.

### Synthesis of CAF01

CAF01 was synthesized according to the methods published by Davidsen et al.^92^. TDB (InvivoGen, San Diego, CA, USA) and dimethyldioctadecylammonium bromide salt (DDA; Avanti Polar Lipids, Alabaster, AL, USA) were dissolved in chloroform/methanol (9:1, v/v) and combined in a 4 mL (1 Dram) vial. The organic solvent was dried off using a gentle stream of nitrogen while rotating the vial at an angle to produce a thin film around the bottom quarter of the vial. Vesicles were formed by hydrating the thin film for 20 min at 60 °C in 10 mM TRIS buffer at pH 7.4 followed by manual swirling. The final CAF01 contains TDB and DDA at a molar ratio of 8.09:1 in 10 mM TRIS buffer.

### Human PBMC isolation and stimulation

Human PBMCs were isolated from peripheral blood of healthy volunteers using gradient centrifugation as described previously^93^. Blood draws were approved by the University of Montana Institutional Review Board and written informed consent was given by all donors prior to blood draws. PBMCs were resuspended in RPMI-1640 media containing 5% autologous plasma and seeded in 96-well flat bottom plates (Thermo Fisher Scientific, Waltham, MA, USA) at 5 × 10^5^ cells/well, followed by stimulation with 5, 10, 25, 50 µM UM-1098/A-SNP, blank A-SNPs volume matched to the highest dose of UM-1098, 5 or 50 µM TDB formulated in CAF01. Unstimulated cells received media only. Cells were incubated for 24 h at 37 °C, 5% CO_2_, centrifuged (500 g, 5 min, RT) and supernatants were harvested and stored at −20 °C. Cytokine analysis was performed in supernatants using MesoScale Discovery (MSD) U-PLEX Assay Platform (MesoScale Diagnostics, Rockville, MD, USA) according to the manufacturer’s instructions. In order to display samples that had a cytokine value of 0 pg/ml on a log scale, all 0 values were arbitrarily set to 0.01 prior to graphing and statistical analysis. Data was analyzed by Ordinary one-way ANOVA with Tukey’s or Dunnett’s multiple comparison test.

### Viability assay

The viability of hPBMCs after stimulation with UM-1098/A-SNP, TDB or CAF01 was assessed using CellTiter-Glo® Luminescent Cell Viability Assay (Promega, Madison, WI, USA) according to the manufacturer’s instructions. Briefly, after having removed supernatants for cytokine analysis, cell pellets were lysed and the amount of ATP was measured via luminescence. Luminescence values for unstimulated cells were regarded as 100% and used to determine the percentage of live cells in all other samples.

### Murine studies

Mice were bred under specific pathogen-free conditions and housed in an AAALAC-accredited facility at the University of Montana in Missoula or Washington University in St. Louis. Studies were performed in accordance with National and Institutional guidelines for animal care under Institutional Animal Care and Use Committee (IACUC) approved protocols.

Male and female six- to eight-week old C57BL/6, BALB/c and C57BL/6 Mincle KO mice were used for in vivo studies. C57BL/6 and BALB/c wt breeder mice were obtained from the Jackson Laboratory (JAX®) and bred in house either at University of Montana in Missoula or Washington University in St. Louis. Mincle KO mice were obtained cryopreserved from Mutant Mouse Resource & Research Centers (MMRRC) at University of California Davis, (strain name: C57BL/6-Clec4etm1.1Cfg/Mmucd, catalogue no. 031936-UCD) and bred at the University of Montana. Absence of the *clec4* gene in Mincle KO mice was confirmed via PCR (Supplementary Fig. 2). Briefly, DNA was extracted from ear punches from C57BL/6 wt and Mincle KO mice and subjected to PCR according to a protocol developed by MMRRC, University of California Davis. Primers used for PCR were 31936-P1 (ATTGCCACTGACCCTCCACC), 31936-P2 (CCCCTGTCACTGTTTCTCTGCA), 31936-P3 (TGCAGCCCAAGCTGATCCTC) and the PCR protocol was as follows: Initiation (94 °C, 5 min), 40 cycles of: denaturation (94 °C, 15 s), annealing (65 °C for first 10 cycles and 55 °C for next 30 cycles), elongation (72 °C 40 sec), final amplification (72 °C, 5 min). PCR products for wt mice have a size of 593 bp and products for KO mice have a size of 488 bp.

### Mouse PBMC isolation and stimulation

PBMCs were isolated from whole blood from adult C57BL/6 and BALB/c wt mice using gradient centrifugation. Blood was obtained via cardiac puncture and used pooled from 5 – 10 mice per experiment. Whole heparinized blood was diluted 1:1 with RT Dulbecco’s Phosphate-Buffered Saline (DPBS) prior to layering onto Lympholyte® Mammal Cell Separation Media (Cedar Lane Laboratories, Burlington, Ontario, Canada). Cells were spun at 800 g for 20 min at RT. Buffy coat layers were collected and washed two times (800 g, 10 min, RT) with DPBS containing 5% heat inactivated fetal bovine serum (HI-FBS). Cells were resuspended in RPMI 10% HI-FBS and seeded into sterile 96-well flat bottom plates at 1 ×10^6^ cells/well. Cells were immediately stimulated with 5 or 50 µM UM-1098 A-SNP 200 or blank A-SNP 200 volume matched to the 50 µM UM-1098 dose. Unstimulated cells received media only. After incubation at 37 °C for 24 h with 5% CO_2_, cells were spun (500 g, 5 min, RT) and supernatants were frozen at −20 °C until analysis. Secreted cytokines in cell supernatants were analyzed by MSD U-PLEX Assay Platform (MesoScale Diagnostics, Rockville, MD, USA) following the manufacturer’s instructions. Data was analyzed by Ordinary one-way ANOVA with Tukey’s multiple comparison test.

### UM-1098 I.V. injection and innate cytokine profiling in mice

Adult C57BL/6 and BALB/c mice (seven animals/group) were injected intravenously (I.V.) with 250 nmol UM-1098/A-SNP, or volume matched blank A-SNPs or vehicle (2% glycerol) while under isoflurane anesthesia. Mice were bled via submandibular cheek bleed 4 h post injection for collection of serum and euthanized 24 h post injection when blood was collected via cardiac puncture. Mice were monitored for toxicity for 1 continuous hour after I.V. administration of UM-1098 as well as at 24 h post injection. Sera were analyzed for innate cytokines IL-6, IL-12p70, IL-23, IL-1β, TNF-α, IL-4, MIP-1α using MSD U-PLEX Assay Platform (MesoScale Diagnostics, Rockville, MD, USA) as per the manufacturer’s instructions. In order to display samples that had a cytokine value of 0 pg/ml on a log scale, all 0 values were arbitrarily set to 0.01 prior to graphing and statistical analysis. Data was analyzed by Ordinary one-way ANOVA with Tukey’s multiple comparison test.

### Mouse vaccination studies

Mice were vaccinated three times intramuscularly, either two or four weeks apart and sacrificed three weeks after the last booster. Vaccinations were given as 50 µl in the left gastrocnemius muscle. For the first in vivo study (Figs. 7, 8), C57BL/6 wt, C57BL/6 Mincle KO and BALB/c wt mice were used. Three to five mice per strain were either vaccinated with 1 µg M72 alone, 1 µg M72 + 50 nmol UM-1098 A-SNP 200 or 1 µg M72 + blank A-SNPs volume matched to UM-1098/A-SNP. Unvaccinated Mincle KO mice served as naïve controls to test for unexpected responses to antigen re-stimulation or underlying antigen specific antibody. All subsequent in vivo studies included naïve C57BL/6 wt mice as controls to determine baseline values of Th cell cytokines and antibody. For the second in vivo study (Figs. 9, 10), only C57BL/6 wt mice were used. For the experiments shown in Figs. 9 and 10, ten mice per group were either vaccinated with antigen alone (M72 or ESAT6/Ag85B), antigen plus 50 nmol UM-1098/A-SNP, blank A-SNPs volume matched to UM-1098 or remained unvaccinated. M72 was used at 1 µg/injection, ESAT6 and Ag85B were used together at 0.1 µg and 0.5 µg respectively. ESAT6 and Ag85B were provided by BEI Resources (Manassas, VA, USA) obtained under National Institutes of Health (NIH) contract AI-75320. Recombinant M72 was either obtained from GlaxoSmithKline (GSK) or recombinantly expressed in *E. coli* BL21 (DE3) pLysS and purified using Ni+ NTA affinity chromatography at Washington University in St. Louis. Vaccines were diluted in 2% glycerol in sterile water and incubated by end-over-end mixing for 60–90 min at RT prior to injection to allow for efficient antigen adsorption to A-SNPs. Mice were monitored for signs of adverse effects for 15 min continuously as well as at 1 h and 24 h after each injection. Serum was collected by cardiac puncture 21 d post-tertiary injection. Draining inguinal and popliteal lymph nodes from the left hind leg and spleens were collected 21 d post tertiary injection for re-stimulation with antigen. Single cell suspensions were obtained from lymph nodes and spleens by mechanical disruption as described previously^93,94^.

### Antigen re-stimulation and cytokine analysis

Single cell suspensions of spleens and draining lymph nodes were re-stimulated with 1 µg/ml M72 or 1 µg/ml of each ESAT6 and Ag85B used in combination for 72 h at 37 °C, 5% CO_2_ in 96-well moat plates (Thermo Fisher Scientific). Supernatants were collected by centrifugation (500 g, 5 min, RT) and stored at −20 °C. Supernatants were analyzed for IL-17A, IFN-γ, IL-5 and TNF-α using MSD U-PLEX Assay Platform (MesoScale Diagnostics, Rockville, MD, USA) as per the manufacturer’s instructions. To display samples that had a cytokine value of 0 pg/ml on a log scale, all 0 values were arbitrarily set to 0.01 prior to graphing and statistical analysis. Additionally, intracellular cytokines were analyzed in splenocytes using flow cytometry: One million cells per sample were incubated with 1 µg/ml antigen (M72 or ESAT6/Ag85B), 1 µg/ml anti-mouse CD28 (BD Biosciences, Franklin Lakes, NJ, USA) and 1 µg/ml anti-mouse CD49d (BD Biosciences, Franklin Lakes, NJ, USA) for 6 h at 37 °C 5% CO_2_ in 96-well plates prior to addition of 1 µl/ml GolgiPlug^TM^ (BD Biosciences, Franklin Lakes, NJ, USA). After addition of GolgiPlug^TM^, cells were incubated for another 12 h, centrifuged, washed, blocked with anti-mouse CD16/CD32 (mouse Fc block, BD Biosciences, Franklin Lakes, NJ, USA) for 10 min at RT and stained (30 min, dark, on ice) for flow cytometric analysis with the following antibodies all acquired from Tonbo Biosciences (San Diego, CA, USA): anti-mouse CD3e PerCP-Cy5.5 (# 65-0031), anti-mouse CD8a PE-Cy7 (# 60-0081-U100), anti-mouse CD4 APC-Cy7 (# 25-0042-U100). Cells were simultaneously stained for viability using 1 µl/sample GhostRed 710 (# 13-0871-T100). For intracellular cytokine staining, cells were first fixed and permeabilized using Cytofix/Cytoperm^TM^ (BD Biosciences, Franklin Lakes, NJ, USA) as per the manufacturer’s instructions. Intracellular cytokine staining was performed for 30 min in the dark on ice using the following antibodies: anti-mouse IL-2 FITC (BioLegend # 503806, San Diego, CA, USA), anti-mouse IFN-γ PE-CF594 (BD Biosciences # 562303, Franklin Lakes, NJ, USA), anti-mouse IL-17A PE (BioLegend # 506904 San Diego, CA, USA), anti-mouse TNF-α APC (BioLegend # 506308, San Diego, CA, USA), anti-mouse IL-5 BV421 (BD Bioscienses # 554391, Franklin Lakes, NJ, USA). Data was collected using an LSRII flow cytometer (BD Biosciences, Franklin Lakes, NJ, USA) and analyzed using FlowJo 10.0 software. Cells were gated for singlets, cells, live/CD3 + , CD4 + /CD8- cells and finally the individual cytokines IFN-γ, IL-17A, IL-2, IL-5, TNF- α (Supplementary Fig. 5).

### *Mtb* challenge

Male and female C57BL/6 wt mice (five animals/group) were vaccinated I.M. three times two weeks apart with either M72 or ESAT6/Ag85B + 50 nmol UM-1098/A-SNP as described above. Animals vaccinated with volume matched blank A-SNPs served as controls. At the time of the 3^rd^ vaccination, one previously unvaccinated group of mice was immunized subcutaneously (S.C.) with 10^6^ CFU/0.2 ml of BCG (BCG Pasteur, Source: Trudeau Institute)^95^. *Mtb* strain HN878 (BEI Resources, Manassas, VA, USA) was used as the challenge strain. It was grown to mid-log phase in Proskauer Beck medium containing 0.05% Tween80 and frozen in 1 ml aliquots at −80 °C^95^. Thirty days after the last booster, mice were challenged by aerosol with a low dose (100 CFU) of *Mtb* HN878 as previously described^96^. Mice were sacrificed 30 d post challenge by CO_2_ asphyxiation, and the lungs were aseptically excised and individually homogenized in physiological saline solution. Serial dilutions of lung homogenates were plated on 7H11 agar for CFU determination and counted after 3 weeks of incubation at 37 °C as described before^95^. Lung inflammation was assessed as area occupied by inflammatory cell infiltrates in Formalin-Fixed Paraffin-Embedded (FFPE), hematoxylin and eosin (HE) stained lung sections as described previously^97^.

### ELISA for detection of antigen specific IgG

96-well Nunc MaxiSorp flat bottom plates (Thermo Fisher Scientific, Waltham, MA, USA) were coated with 1 µg/ml ESAT6 or Ag85B or M72 in DPBS for 24 h at RT. Plates were washed 3x with 1× PBS + 0.05% Tween-20 and blocked with superblock (ScyTek Laboratories, Logan, UT, USA) at 37 °C for 2 h. Sera were used at various starting dilutions ranging from 1:25 to 1:1000 and serially diluted eight times down the plate. Plates were incubated with sera at 37 °C for 1 h followed by five washes. HRP-conjugated goat anti-mouse IgG/IgG1/IgG2c/IgG2a (Southern Biotech, Birmingham, AL, USA) detection antibodies were used at 1:3000 for ESAT6 and Ag85B or at 1:5000 for M72. Antibody against IgG2c was used for sera of C57BL/6 wt and Mincle KO mice and antibody against IgG2a was used for sera of BALB/c mice. After three washes, plates were incubated with 0.1 ml/well 3,3’,5,5’-Tetramethylbenzidine substrate (BD Biosciences, Franklin Lake, NJ, USA) for 15 min followed by addition of 0.05 ml/well 2 N sulfuric acid (RICCA Chemical Company, Arlington, TX, USA) as stop reagent. Finally, plates were read at 450 nm using a SpectraMax 190 microplate reader (Molecular Devices, San Jose, CA, USA). Pooled serum from C57BL/6 mice vaccinated three times with ESAT6/Ag85B + UM-1098/A-SNP derived from a different study was used as a standard for all ESAT6 and Ag85B ELISAs. Pooled serum from mice vaccinated three times with M72 + UM-1098/A-SNP derived from a different study served as standard for all M72 ELISAs. Standard sera were run as eight three-fold serial dilutions on each plate and used to generate a standard curve (Microsoft XLfit). The OD_450_ of the lowest dilution of the standard was arbitrarily set to 1000 ELISA units and XLfit was used to correlate OD_450_ of all serum samples to ELISA units based on the standard curve and taking into consideration the dilutions used. Only OD_450_ values that fell within the linear range of the standard curve and were at least 1.5x the background were used for determination of antigen-specific ELISA units. If OD_450_ values for samples were below the threshold, they were assigned a value of 0. In order to graph 0 values on a log scale, all 0 values were arbitrarily set to 0.01 prior to graphing and statistical analysis.

### Statistical analysis

All data was analyzed using GraphPad Prism 9. All in vitro experiments were repeated at least five times. All measurements were taken from distinct samples. Data was analyzed for normal distribution using the Kolmogorov-Smirnov test. If data were normally distributed, ordinary one-way ANOVA plus Tukey’s or Dunnett’s multiple comparison test was used to determine significant differences between groups. Significant outliers were removed using GraphPad Prism outlier calculator and the Grubbs’ test with alpha = 0.05. Antibody data for the in vivo study comparing C57BL/6, Mincle KO and BALB/c mice was log transformed prior to statistical analysis. All data are displayed as the mean plus standard deviation. Significances were considered as follows: *p < 0.05, ** *p* < 0.01, ***p < 0.001, ****p < 0.0001.

### Reporting summary

Further information on research design is available in the Nature Research Reporting Summary linked to this article.

### Supplementary information

Supplementary Information

Reporting Summary

## Data Availability

All data generated or analyzed during this study are included in this article including its supplementary information files. Additional information is available by contacting the corresponding author (jay.evans@umontana.edu).
