# Supplementary material for: Vaccination with Mincle agonist UM-1098 and mycobacterial antigens induces protective Th1 and Th17 responses
Source: NPJ Vaccines. 2024 Jun 6;9:100. doi: 10.1038/s41541-024-00897-x (PMC11156909; doi:10.1038/s41541-024-00897-x)
Supplement: Supplementary file 1 — Supplementary Information [file 41541_2024_897_MOESM1_ESM.pdf]

Supplementary Information

a

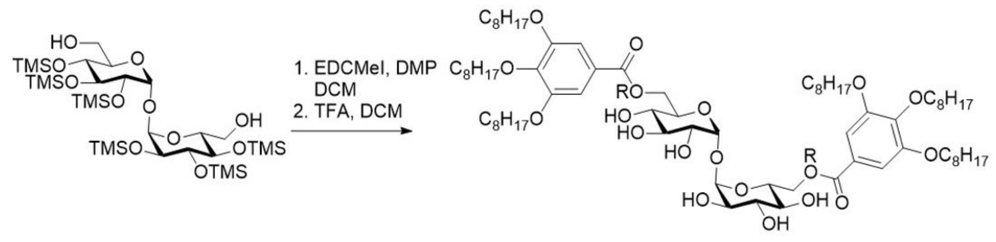

b

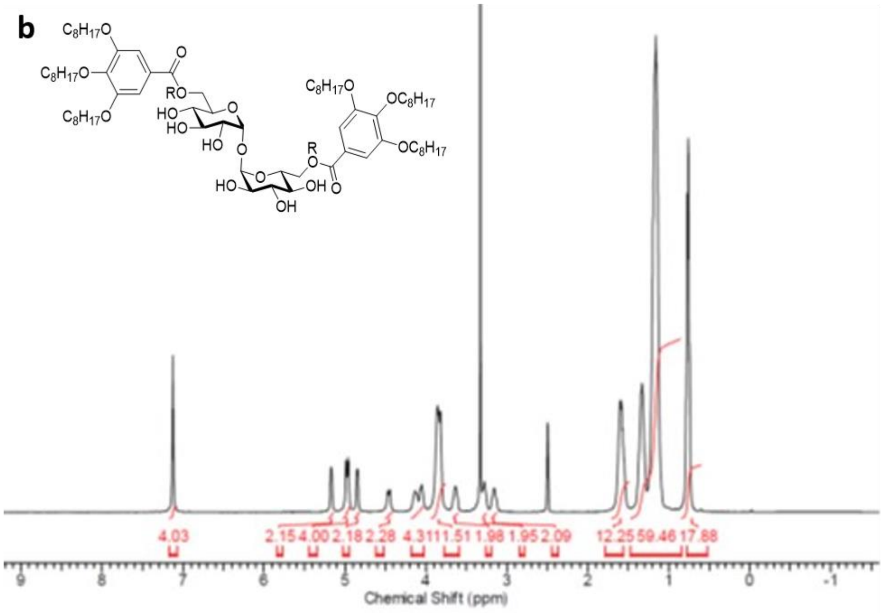

c

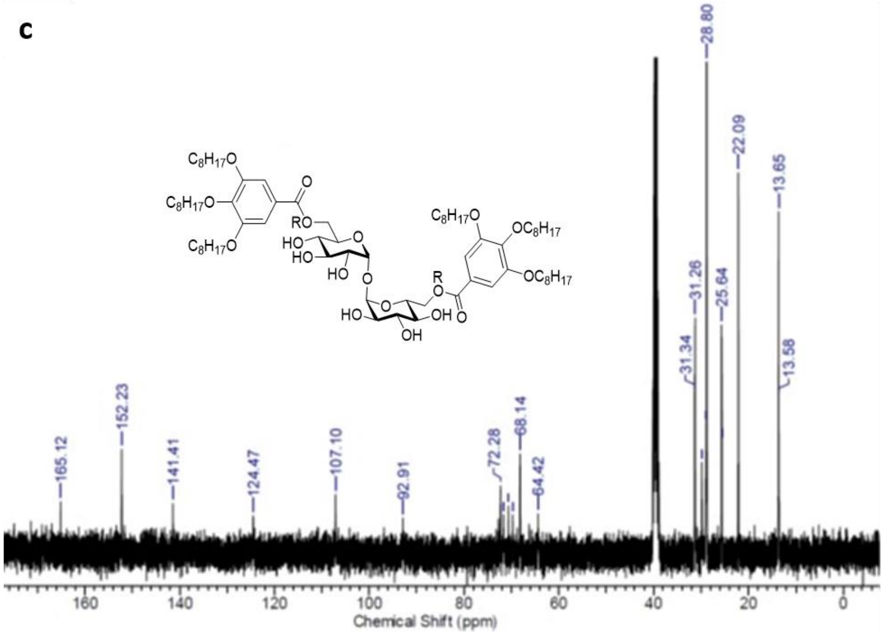

Supplementary Figure 1. Synthesis of UM-1098 (**a**) and ^{1}H NMR (400 MHz, DMSO-d6) and ^{13}C NMR (100 MHz, DMSO-d6) spectra (**b** - **c**) of UM-1098.

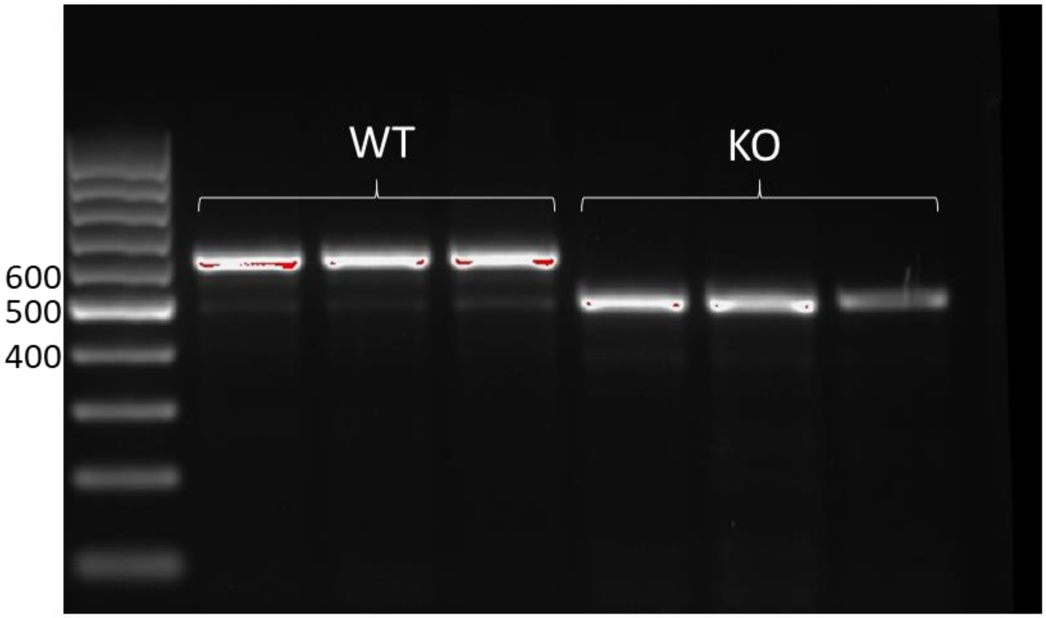

Supplementary Figure 2. Agarose gel electrophoresis image of PCR products confirming absence of the *clec4e* gene in Mincle KO mice.

DNA was extracted from ear punches from C57BL/6 wt and Mincle KO mice and used in PCR with primers detecting the Mincle/*clec4e* gene. PCR products were separated via agarose gel electrophoresis and visualized with UV light. PCR products from C57BL/6 wt DNA (lanes 2-4) have a size of 593 bp and PCR products from Mincle KO DNA (lanes 5-7) have a size of 488 bp. A 100 bp DNA ladder is shown in the first lane.

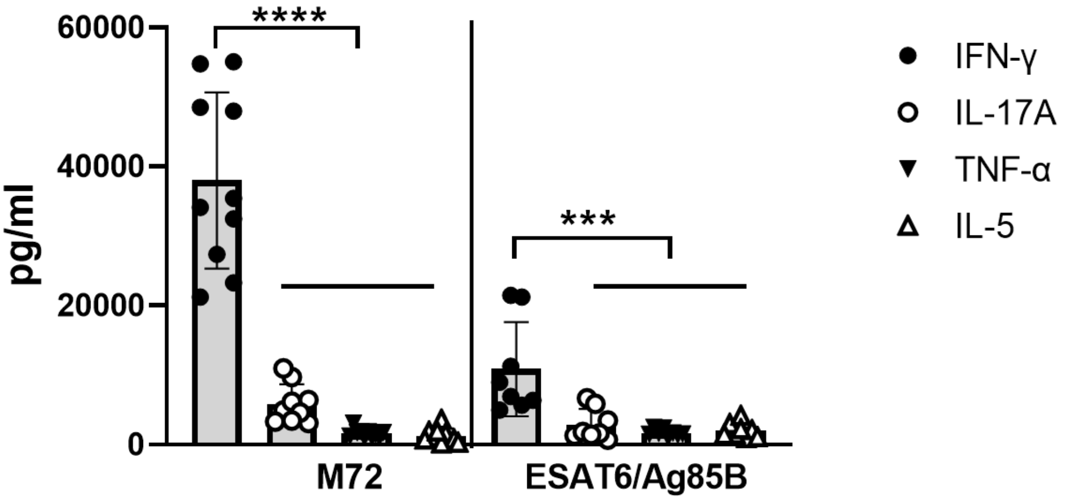

Supplementary Figure 3. Secreted cytokines of antigen re-stimulated draining lymph node cells of mice 21 days post tertiary vaccination with *Mtb* antigens and UM-1098/A-SNP.

Ten mice per group were vaccinated three times, two weeks apart with antigen + UM-1098/A-SNP. Antigens were either M72 or the combination of ESAT6 and Ag85B. Mice were sacrificed three weeks post tertiary vaccination and single cell suspensions of draining lymph nodes were re-stimulated with antigen for 72 h. Cytokines were measured in supernatants using the MesoScale Discovery (MSD) U-PLEX Assay. Data was analyzed by Ordinary one-way ANOVA with Tukey's multiple comparisons test and is represented as the mean and SD. Significances were considered as follows: *p<0.05, ** p<0.01, ***p<0.001, **** p<0.0001.

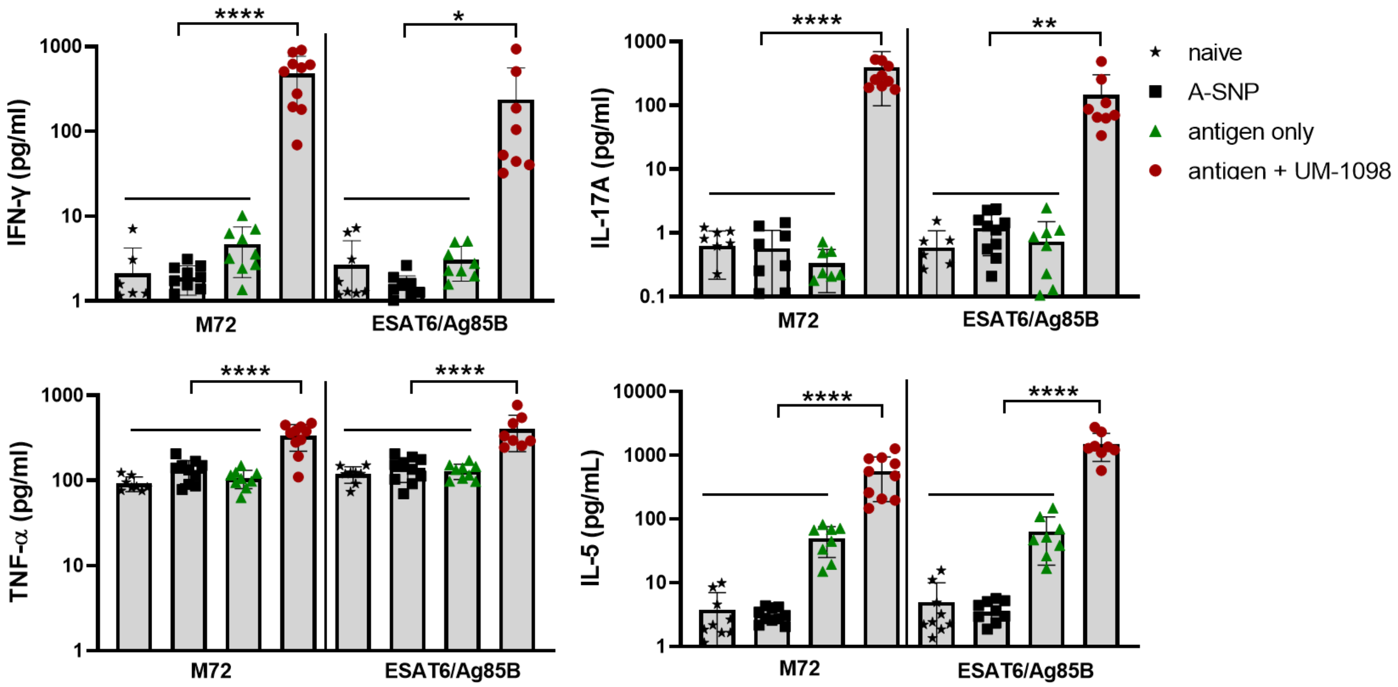

Supplementary Figure 4. Secreted cytokines of antigen re-stimulated splenocytes of mice 21 days post tertiary vaccination with *Mtb* antigens +/- UM-1098/A-SNP.

Mice were vaccinated three times, two weeks apart with either blank A-SNPs, antigen alone, or antigen + UM-1098/A-SNP. Antigens were either M72 or the combination of ESAT6 and Ag85B. Unvaccinated mice served as naïve controls. Mice were sacrificed three weeks post tertiary vaccination and single cell suspensions of splenocytes were re-stimulated with antigen for 72 h. Cytokines were measured in supernatants using the MesoScale Discovery (MSD) U-PLEX Assay. Data is represented on a log scale with each data point representing one animal. Data was analyzed by Ordinary one-way ANOVA with Tukey's multiple comparisons test and is represented as the mean and SD. Significances were considered as follows: *p<0.05, ** p<0.01, ***p<0.001, **** p<0.0001.

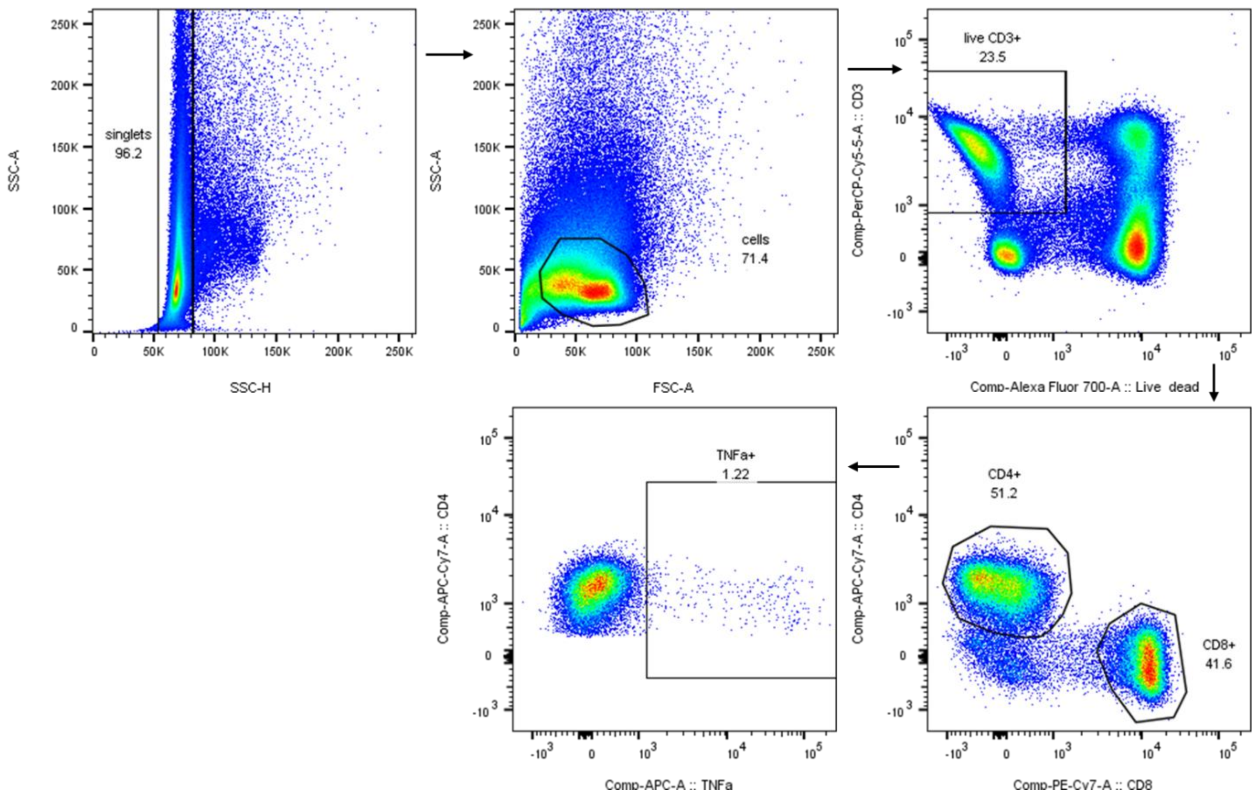

Supplementary Figure 5. Gating strategy for antigen re-stimulated lymphocytes in the spleens of mice 21 days post tertiary vaccination with *Mtb* antigens +/- UM-1098/A-SNP.

Mice were vaccinated three times, two weeks apart with either blank A-SNPs, antigen alone, or antigen + UM-1098/A-SNP. Antigens were either M72 or the combination of ESAT6 and Ag85B. Unvaccinated mice served as naïve controls. Mice were sacrificed three weeks post tertiary vaccination and single cell suspensions of spleens were re-stimulated with antigen for 18 h in the presence of GolgiPlug™, anti-mouse CD28 and anti-mouse CD49d antibodies. Samples were analyzed by flow cytometry and gated as follows: singlets, cells, live/CD3+, CD4+/CD8- cells and finally the individual cytokines. A representative dot blot for TNF-α is shown above.

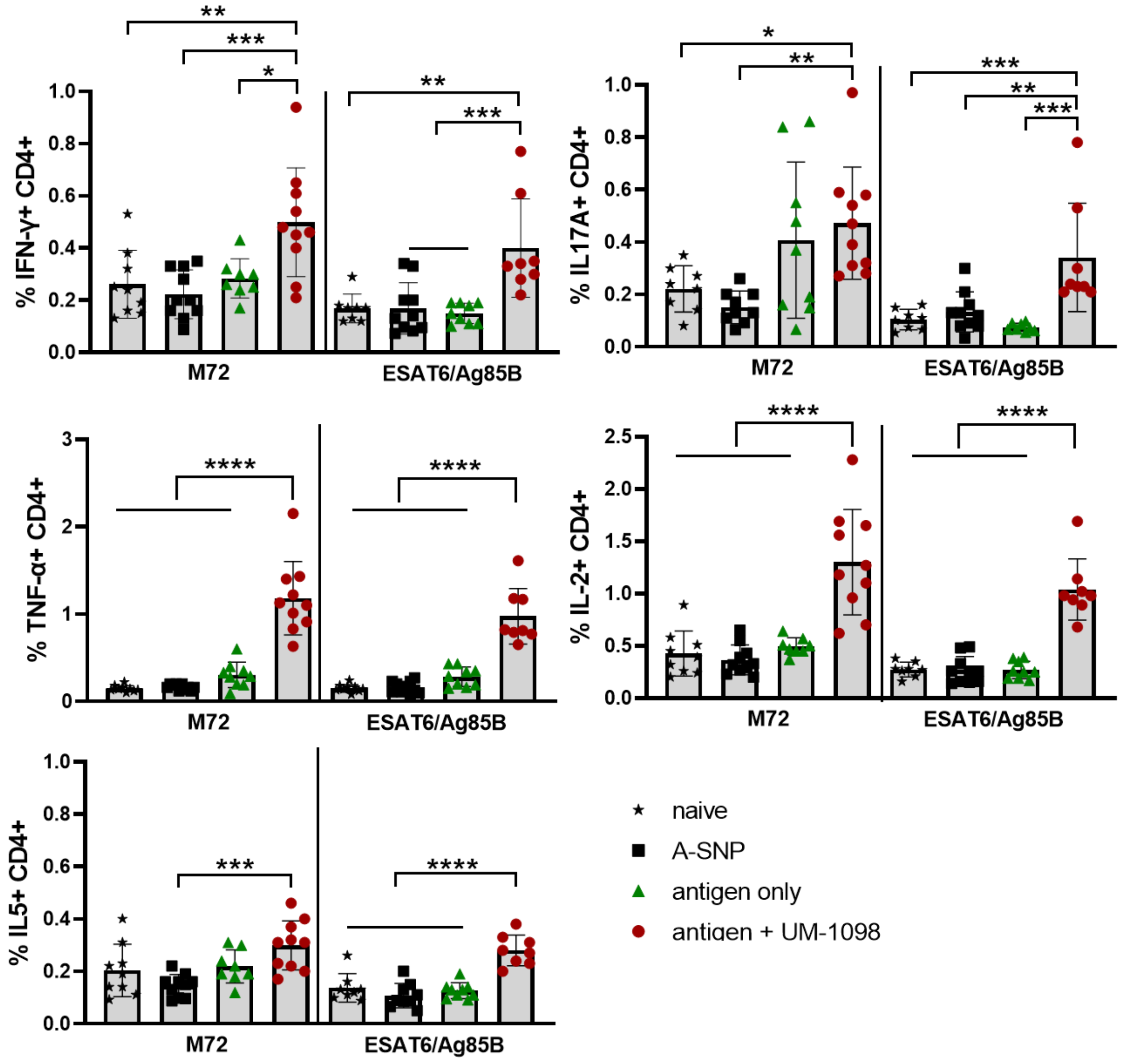

Supplementary Figure 6. Intracellular cytokine staining of antigen re-stimulated CD4+ lymphocytes in the spleens of mice 21 days post tertiary vaccination with *Mtb* antigens +/- UM-1098/A-SNP.

Mice were vaccinated three times, two weeks apart with either blank A-SNPs, antigen alone, or antigen + UM-1098/A-SNP. Antigens were either M72 or the combination of ESAT6 and Ag85B. Unvaccinated mice served as naïve controls. Mice were sacrificed three weeks post tertiary vaccination and single cell suspensions of spleens were re-stimulated with antigen for 18 h in the presence of GolgiPlug™, anti-

mouse CD28 and anti-mouse CD49d antibodies. Samples were acquired by flow cytometry and analyzed by FlowJo 10.0 software followed by Ordinary one-way ANOVA with Tukey's multiple comparisons test. Data is shown as the mean and standard deviation of % cytokine positive CD4+ cells. Significances were considered as follows: *$p<0.05$, ** $p<0.01$, ***$p<0.001$, **** $p<0.0001$.

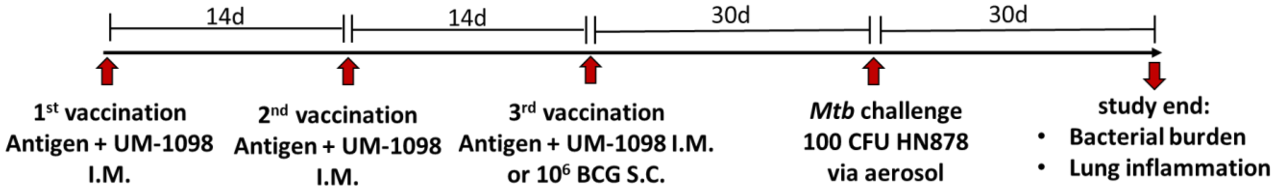

Supplementary Figure 7. Timeline of *Mtb* challenge in mice.

Mice were either vaccinated I.M. three times, two weeks apart with antigen + UM-1098/A-SNP or blank A-SNPs or one-time S.C. with 10^6 CFU of BCG (n = 5 mice/group). Mice were challenged with 100 CFU of *Mtb* HN878 via aerosol 30 days after the last booster and sacrificed 30 days post infection for determination of lung inflammation and bacterial burden in lungs.

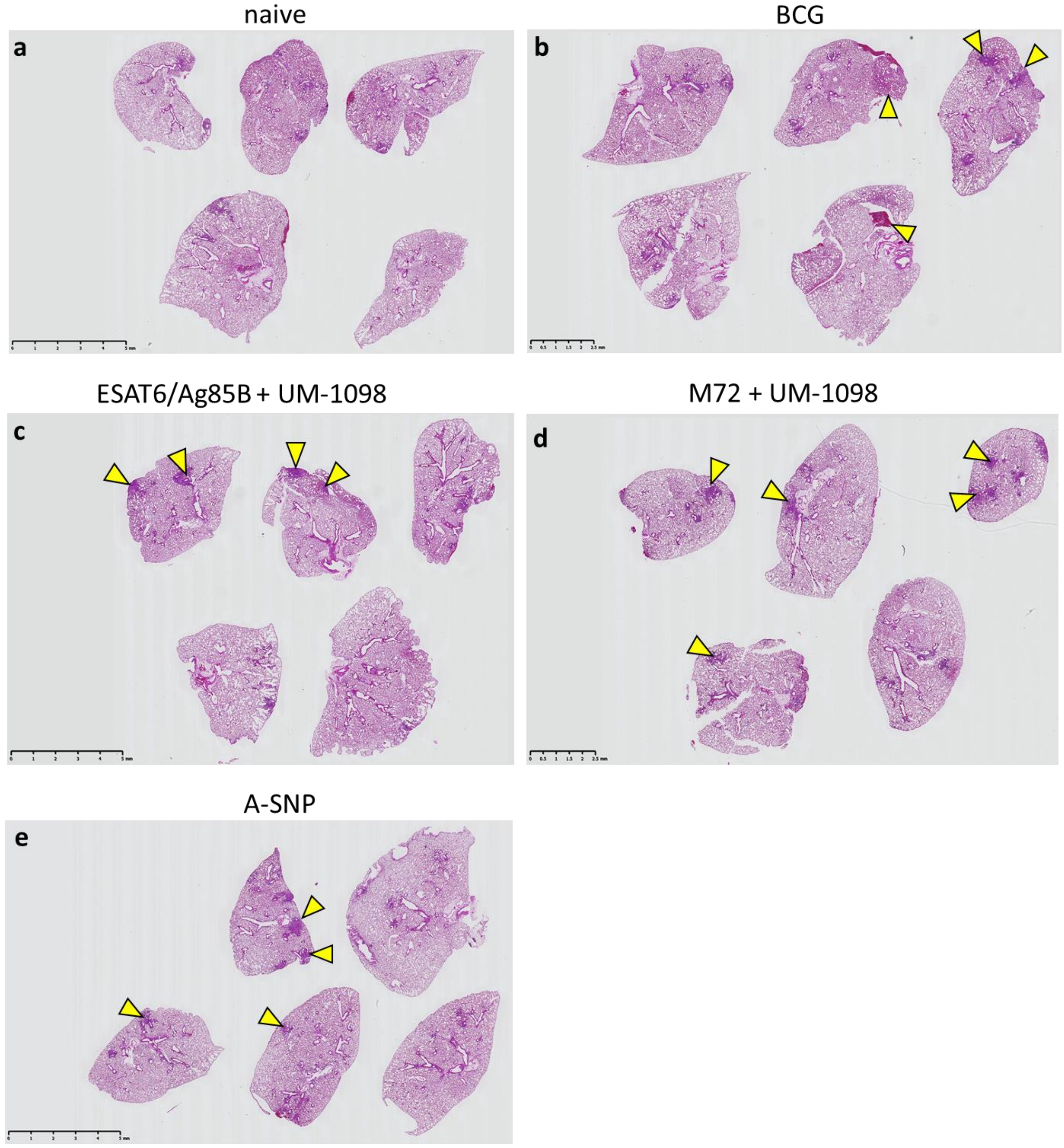

Supplementary Figure 8. Histological images of lungs from mice 30 days post *Mtb* challenge.

Five mice per group were either unvaccinated (**a**), vaccinated with BCG (**b**), ESAT6/Ag85B + UM-1098/A-SNP (**c**), M72 + UM-1098/A-SNP (**d**) or blank A-SNPs (**e**). Lungs were formalin fixed, paraffin embedded

and stained with hematoxylin and eosin. Each mouse is represented with one lobe. Arrowheads represent areas of cell infiltrates. The scale bar ranges from 0 to 5mm in 1mm increments (**a, c, e**) or from 0 to 2.5mm in 0.5mm increments (**b, d**).

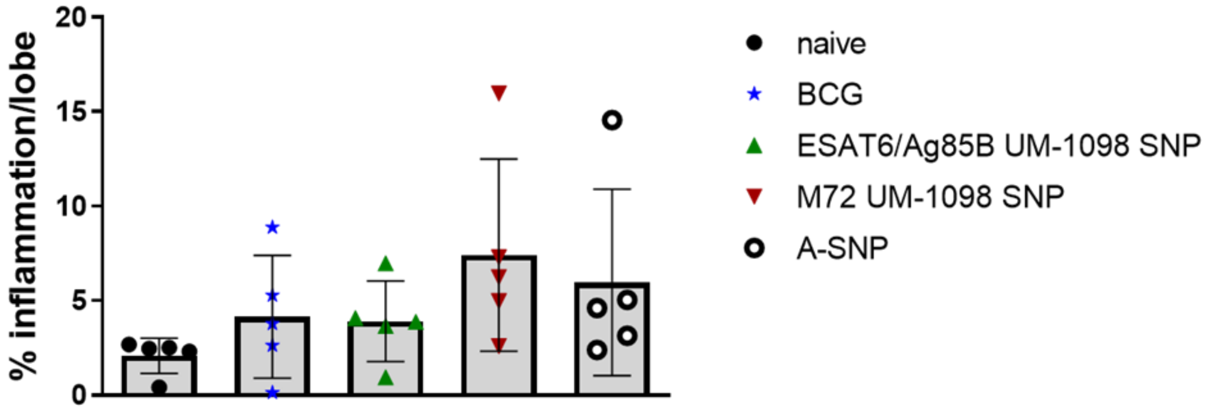

Supplementary Figure 9. Pulmonary inflammation in mice 30 days after challenge with *Mtb* HN878.

Vaccination with UM-1098 adjuvanted M72 or ESAT6/Ag85B does not induce significant lung inflammation in mice 30 days after challenge with *Mtb* HN878. Five C57BL/6 mice per group were vaccinated I.M. with UM-1098/A-SNP adjuvanted M72 or ESAT6/Ag85B or blank A-SNPs three times two weeks apart. Unvaccinated or BCG vaccinated mice served as controls. Inflammation was assessed by measuring cell infiltrates by histology and is shown as % inflammation/lobe. Data was analyzed by Ordinary one-way ANOVA with Tukey's multiple comparisons test and is represented as the mean and SD. Significances were considered as follows: *p<0.05, ** p<0.01, ***p<0.001, **** p<0.0001. No significant differences between groups were found.
